# Common and Specific Characteristics of Adolescent Bipolar Disorder Types I and II: A Combined Cortical Thickness and Structural Covariance Analysis

**DOI:** 10.3389/fpsyt.2021.750798

**Published:** 2022-01-21

**Authors:** Liangfeng Kuang, Weijia Gao, Zhiliang Long, Weifang Cao, Dong Cui, Yongxin Guo, Qing Jiao, Jianfeng Qiu, Linyan Su, Guangming Lu

**Affiliations:** ^1^Department of Radiology, Shandong First Medical University & Shandong Academy of Medical Sciences, Taian, China; ^2^Department of Child Psychology, The Children's Hospital, Zhejiang University School of Medicine, Hangzhou, China; ^3^Faculty of Psychology, Southwest University, Chongqing, China; ^4^Institute of Biomedical Engineering, Chinese Academy of Medical Science and Peking Union Medical College, Tianjin, China; ^5^Mental Health Institute of The Second Xiangya Hospital, Central South University, Changsha, China; ^6^Department of Medical Imaging, Jinling Hospital, Clinical School of Medical College, Nanjing University, Nanjing, China

**Keywords:** bipolar disorder, MRI, neuroimaging, cortical thickness, structural covariance, subtype

## Abstract

**Background:**

By calculating cortical thickness (CT) and cortical structural covariance (SC), we aimed to investigate cortical morphology and cortical inter-regional correlation alterations in adolescent bipolar disorder type I (BD-I) and type II (BD-II) patients.

**Methods:**

T1-weighted images from 36 BD-I and 22 BD-II patients and 19 healthy controls (HCs) were processed to estimate CT. CT values of the whole brain were compared among three groups. Cortical regions showing CT differences in groups were regarded as seeds for analyzing cortical SC differences between groups. The relationship between CT and clinical indices was further assessed.

**Results:**

Both BD groups showed cortical thinning in several frontal and temporal areas vs. HCs, and CT showed no significant difference between two BD subtypes. Compared to HCs, both BD groups exhibited reduced SC connections between left superior frontal gyrus (SFG) and right postcentral gyrus (PCG), left superior temporal gyrus (STG) and right pars opercularis, and left STG and right PCG. Compared with HCs, decreased SC connections between left STG and right inferior parietal gyrus (IPG) and right pars opercularis and right STG were only observed in the BD-I group, and left PCG and left SFG only in the BD-II group. CT of right middle temporal gyrus was negatively correlated with number of episodes in BD-II patients.

**Conclusions:**

Adolescent BD-I and BD-II showed commonly decreased CT while presenting commonly and distinctly declined SC connections. This study provides a better understanding of cortical morphology and cortical inter-regional correlation alterations in BD and crucial insights into neuroanatomical mechanisms and pathophysiology of different BD subtypes.

## Introduction

Bipolar disorder (BD) is a common mental disorder featuring violent emotional fluctuations (1). BD can be divided into two subtypes, type I (BD-I) and type II (BD-II). BD-I is characterized by at least one full-blown manic episode, and BD-II is defined as alternating episodes of depression and hypomania. The disorder is associated not only with premature death, severe disability, and psychosocial impairment (2), but also with impaired cognition (3). The diagnosis of BD is mainly through clinical history, interview, and behavioral observations, which may lead to misdiagnosis, improper treatment, and eventually poorer outcomes (4). So far, the pathophysiology underlying BD is still unclear. Fortunately, brain imaging provides a non-invasive approach for exploring the brain structure, making it a hot spot in the study of the pathological mechanism of mood disorders (5). Anatomical brain abnormalities in BD are confirmed by imaging studies, which may help in the development of neuroimaging markers (6). For better understanding the underlying mechanisms of BD, investigating neural structural changes may contribute to improving treatment and diagnosis of individuals with BD. Although the neural underpinnings of BD progression remain unclear, research suggests that mood episodes could cause lasting neurobiological changes (7). Individuals with BD-I and BD-II exhibit different symptoms, which partly indicates different neurobiological mechanisms and pathophysiology of the two BD subtypes (8). However, little is known about common and special structural abnormalities of BD-I and BD-II (9).

Cortical thickness (CT) is beneficial for identifying biological markers of BD, which has been found in prior review (10). CT thinning is a consistent neuroimaging finding in BD patients, primarily in frontal and temporal cortex and then in anterior cingulate and parietal regions (11–16). Clinical evidence shows that the course of disease and related health problems in BD-II patients are as severe as in BD-I patients (1). Nevertheless, the majority of studies in BD focus on individuals with BD-I or a combination of BD-I and BD-II. Thus far, there are relatively few studies paying attention to probing CT changes in BD-I, BD-II patients, and healthy controls (HCs). In these studies, region-of-interest (ROI)–based and data-driven methods were used to analyze CT in some regions and whole brain areas, respectively. In an ROI-based study, combining BD in adolescents and young adults, uncorrected results show that BD-II patients had reduced CT in anterior cingulate cortex compared with BD-I patients (17). Using data-driven methods, a study reports that both adult BD-I and BD-II shared lower CT in several frontal regions, but only the BD-I group showed thinner cortices in medial prefrontal and temporal regions relative to HCs (8). However, a recent study, also employing a data-driven approach, focuses on adult (age ≥25 years) and adolescents/young adults (age <25 years) with BD and reports no significant differences in CT between BD-I and BD-II groups, and whole adult BD subjects showed CT reductions in left par opercularis, left fusiform gyrus, and left rostral middle frontal gyrus (MFG) compared with HCs, whereas whole adolescent/young adult subjects with BD presented cortical thinning in right supramarginal gyrus (18). Taken together, although these findings of CT alterations in two subtypes of BD remain controversial, these alterations exert an effect on neural processing in BD (19). Hence, it is meaningful to understand the pathophysiology of BD by characterizing thickness of cortex in BD patients. Relative to an ROI-based CT analytic method, data-driven analysis of CT is beneficial for comprehensive understanding of cortical structure alterations. The whole-brain CT alterations of adolescent BD-I and BD-II were needed for further investigation.

In recent years, cortical morphology study is not only limited to CT of one brain region, but also attracted growing interest in cortico-cortical correlations. Structural covariance (SC) describes a phenomenon by which gray matter properties of one brain area may change together with those of other widely distributed cortical areas (20). It could uncover intracortical similarities and be described as the coordinated change in brain morphological measurements (e.g., cortical thickness) (21), showing partial agreement with functional connectivity (22) and white matter connections (23). Recently, an increasing number of studies have confirmed the alterations of cortical SC in various mood disorders (24–26). However, only a few studies investigate cortical SC in individuals with BD. Comparing cortical SC between schizophrenia and BD-I patients, study observes that BD patients presented decreased cortical SC between left superior occipital gyrus and its neighbor anatomic structures and right insular cortex and its adjacent anatomic regions (27). One study compared SC in nucleus accumbens (NAc) in BD and major depressive disorder (MDD) and reports that, compared with HCs, BD groups displayed decreased volumetric SC connections between NAc and prefrontal gyrus, bilateral anterior insula, and bilateral striatum and increased NAc connections between NAc and left hippocampus extending to thalamus (28). When comparing cortical and subcortical volumetric SC in BD and MDD, another study found BD patients showed distinct anatomical relationships of the connections between striatum and dorsolateral prefrontal cortex (DLPFC) and putamen and caudate nuclei vs. HCs (29). In summary, the above evidence accumulated supports the crucial role of structural SC in BD. The SC of brain regions could be interpreted to support the existence of coordinated neurodevelopmental and maturational alteration in distributed cortical regions (20). Given that adolescence is a period for the onset and coordinated neurodevelopment of mental disorders (30), abnormal cortical SC may play a vital role in the pathophysiology of psychiatric disorders. Thus, in brain regions showing CT alterations, evaluating cortical SC abnormalities in adolescent BD-I and BD-II may offer novel insights given that brain cortical regions do not function in isolation.

Herein, we sought to analyze whole-brain CT differences among the adolescent BD-I, BD-II, and HCs and seed-based cortical SC differences. Specifically, CT values of whole brain were compared among the three groups and subgroups. Subsequently, cortical regions showing between-group CT differences were regarded as seeds. Correlation coefficients between each seed and other cortical regions were compared between groups. In addition, we also analyzed the relationship between cortical region thickness and clinical index. According to previous evidence, we hypothesized that patients with BD-I and BD-II would show great CT reductions in frontal and temporal regions compared with HCs and that cortical SC connections among frontal, temporal, and parietal regions would be abnormal in BD-I and BD-II groups.

## Materials and Methods

### Participants

The subjects enrolled were 77 adolescents (BD-I patients, *N* = 36; BD-II patients, *N* = 22; HCs, *N* = 19). All BD patients were outpatients from the clinical psychiatric department in the Second Xiangya Hospital of Central South University, and HCs were recruited by advertisements in local schools. All the BD patients met the criteria of Diagnostic and Statistical Manual for Mental Disorders, Fourth Edition (DSM-IV). The inclusion criteria for all subjects were (a) 12–18 years old, (b) right-handed, and (c) able to hold their head still to finish the MRI scanning. Exclusion criteria of all participants were (a) score of full-scale intelligence quotient (IQ) ≤ 80; (b) pregnancy; (c) contraindications of MRI scanning, including claustrophobia or foreign metallic substances in the body; (d) history of alcohol or drug abuse; (e) history of electroconvulsive therapy; (f) other psychiatric disorders, such as autism, schizophrenia, bulimia nervosa or anorexia, and learning disabilities; (g) active medical or neurological diseases. In addition, HCs were required to be without a history of mental disorder in their first-degree relatives. The study was approved by the Ethics Committee of the Second Xiangya Hospital of Central South University. Informed consent documents were obtained from all participants and at least one legal guardian.

### Demographic and Clinical Evaluation

The diagnoses were determined through consensus between two broad-certified child psychiatrists based on clinical interviews and administrations of the Schedule for Affective Disorders and Schizophrenia for School-Age Children-Present and Lifetime Version (K-SADSPL) (31). Demographic and clinical information of all subjects were collected on the day of MRI scanning. Intellectual ability of all the subjects was assessed by the Wechsler Abbreviated Scale of Intelligence (WASI). The Young Mania Rating Scale (YMRS) (32) and Mood and Feelings Questionnaire (MFQ) (33) were adopted to assess the current mood state of every participant. The information of onset age, illness duration, number of episodes, psychotic symptom, familial BD history, medications, and comorbidity of patients were collected. Four BD patients were in the first episode. Twenty-three patients had not been treated with medication.

IBM SPSS (version 25.0, Armonk, NY, United States) was used for statistical analysis of demographic and clinical data. Categorical variables were compared by Pearson chi-square test. Continuous variables were compared by one-way analysis of variance among the three groups and two-sample *t*-test between two groups. Statistical power was calculated by G^*^Power (version 3.1) (https://www.psychologie.hhu.de/arbeitsgruppen/allgemeine-psychologie-und-arbeitspsychologie/gpower.html).

### MRI Data Acquisition

Siemens 3 Tesla Trio scanner (Siemens, Munich, Germany) was used for MRI scanning. T1-weighted images were acquired by employing a three-dimensional magnetization-prepared rapid acquisition gradient echo (3D MPRAGE) protocol. Sequence parameters were the following: repetition time = 2,300 ms, echo time = 2.03 ms, inversion time = 900 ms, slice thickness = 1 mm, field of view (FOV) = 256 × 256 mm^2^, matrix = 256 × 256, flip angle = 9°, voxel size = 1 × 1 × 1 mm^3^, and 176 axial slices. During MRI scanning, a foam pad placed on two sides of each subject's head was to restrict the subjects' head motion and cotton earplugs reduced the noise and protected subjects' hearing.

### MRI Data Processing

The cortical reconstruction and segmentation were automatically finished employing a FreeSurfer version 6.0 software (https://surfer.nmr.mgh.harvard.edu) longitudinal stream. In brief, this process mainly included motion correction, automated Talairach transformation, intensity normalization, skull stripping, reconstruction of the white and gray matter boundary and the cortical surface, estimation of brain surfaces and surface segmentation. CT values were extracted from 68 (34 regions per hemisphere) cortical gray matter regions based on Desikan–Killiany atlas (34). Cortical reconstructions and segmentations of all subjects were visually inspected by experienced researchers and manually corrected if needed.

### Cortical Thickness Analysis

Age and gender were included as covariates in all comparisons of CT. Analysis of covariance (ANCOVA) was used to analyze CT changes among the three groups. The main effects comparisons of CT were corrected by false discovery rate (FDR) correction (35). For the cortical regions showing significant CT differences among the three groups after FDR correction, *post hoc* comparisons were further conducted with FDR correction for multiple comparisons. Because the relationship between total intracranial volume (TIV) and CT is controversial (36), TIV was not regarded as a covariate in CT analysis. Moreover, using analysis of covariance with age and gender as covariates, no significant difference (*F* = 0.504, *p* = 0.606, Power = 0.137) of TIV was found among the three groups.

### Structural Covariation Analysis

SC analysis between subgroups was conducted employing a seed-based approach on the basis of *post hoc* comparison results from CT analysis. If cortical regions showed significant intergroup differences of CT between groups (BD-I vs. HCs, BD-II vs. HC, BD-I vs. BD-II), these cortical regions were regarded as seeds to analyze the intergroup difference of SC. According to previous study of SC (37), the detailed analysis steps of SC were as follows. First, to construct seed-base cortical SC network of each population (BD-I, BD-II, HCs), partial correlation analysis was separately used to calculate the CT correlation coefficient between each seed and all other cortices with age and gender as covariates. Second, a partial correlation coefficient *r* value was Fisher-transformed to a *z* value as follows:


$$\begin{array}{l} Z_{i}=\frac{1}{2}\;log_{e}\left[\frac{1+r_{i}}{1-r_{i}}\;\right], \end{array}$$

where *r*_*i*_is the partial correlation coefficient *r* value between each seed and each other cortical region, and *i* is each population. Finally, a *z*-test was used to assess intergroup difference of cortical SC network as follows:


$$\begin{array}{l} \text{z}=\frac{Z_{1}-Z_{2}}{\sqrt{\frac{1}{n_{1}-3}+\frac{1}{n_{2}-\;3}}}. \end{array}$$

The sample distribution of *Z*_*i*_ is approximately normal with a variance equal 1/(*n*-3), and *n* refers to the sample size of each population. The standard error of the difference between the two independent *Z*_*i*_ is the square root of that sum of variance, and the standard error is employed as the denominator in the *z*-test. The *z* value was converted to the *p*-value using a normal cumulative distribution function. The *p*-value of this test was corrected using FDR correction at *p* < 0.05.

### Cortical Thickness Correlation Analysis

Exploratory correlation analysis between CT and clinical indices was performed. Pearson correlation analysis was used to analyze the residual relationship between CT and clinical indices with age and gender regressed.

## Results

### Demographic and Clinical Data

As shown in Table 1, three groups did not significantly differ in gender, age, education, and IQ distribution. YMRS and MFQ scores showed significant differences among the three groups. There was no significant difference between BD-I and BD-II groups in onset age, illness duration, number of episodes, first episode, psychotic symptoms, and familial history.

**Table 1:** Demographic and clinical data of all participants.

**Characteristics**	**BD-I (*n* = 36)**	**BD-II (*n* = 22)**	**HCs (*n* = 19)**	**Statistic**	***p***	**Power**
Gender (male/female)	20/16	9/13	7/12	χ^2^ = 2.172	0.338	0.243
Age (years)	15.31 (1.93)	14.64 (1.50)	14.16 (1.57)	*F* = 2.935	0.059	0.535
Education (years)	8.33 (1.90)	7.64 (1.47)	7.47 (2.22)	*F* = 1.667	0.196	0.335
IQ	102.67 (13.60)	105.32 (12.12)	105.32 (7.51)	*F* = 0.471	0.626	0.125
YMRS scores	18.15 (15.25)	9.50 (11.10)	3.63 (2.06)	*F* = 9.690	**<0.001**	0.946
MFQ scores	12.44 (12.30)	15.41 (12.59)	6.11 (3.33)	*F* = 3.849	**0.026**	0.647
Onset age (year)	13.83 (1.81)	13.55 (1.99)	–	*t* = 0.565	0.574	0.085
Illness duration (months)	19.08 (13.33)	11.73 (14.00)	–	*t* = 2.001	0.050	0.497
Number of episodes	3.58 (2.27)	2.91 (1.19)	–	*t* = 1.285	0.204	0.268
Psychotic symptoms (yes/no)	17/19	13/9	–	χ^2^ = 0.770	0.380	0.142
Familial BD history (yes/no)	18/18	11/11	–	χ^2^ = 0.000	1.000	0.050
**Medications**						
Lithium	12	4	–	–	–	–
Valproate	18	8	–	–	–	–
Atypical antipsychotics	22	9	–	–	–	–
Antidepressants	1	1	–	–	–	–
**Comorbidity**						
ADHD	3	1	–	–	–	–
ODD	0	1	–	–	–	–
OCD	3	1	–	–	–	–
Anxiety	2	2	–	–	–	–
TIC	0	1	–	–	–	–

*Values are presented as mean (standard deviation).*

*BD-I, bipolar disorder type I; BD-II, bipolar disorder type II; HCs, healthy subject controls; IQ, intelligence quotient; YMRS, Young Manic Rating Scale; MFQ, Mood and Feelings Questionnaire; ADHD, attention-deficit/hyperactivity disorder; ODD, oppositional defiant disorder; OCD, obsessive–compulsive disorder.*

*χ^2^, value for Pearson chi-square test; F, value for one-way analysis of variance; t, value for two-sample two-tailed t-test.*

*Bold values indicate significant differences with p < 0.05*.

### Cortical Thickness Analysis

As shown in Table 2, the CT of seven cortical regions showed significant differences among the three groups. Compared with HCs, both BD-I and BD II groups exhibited decreased CT in seven cortical regions, involving left inferior temporal gyrus (ITG), left rostral MFG, left superior frontal gyrus (SFG), left transverse temporal gyrus (TTG), right middle temporal gyrus (MTG), and bilateral superior temporal gyrus (STG). There was no significant difference of CT between BD-I and BD-II groups. The results of all CT analyses are shown in the Supplementary Table S1.

**Table 2:** Significant cortical thickness differences among BD-I, BD-II patients and HCs.

										***Post hoc*** **comparisons^b^**					
**Brain region thickness**	**BD-I**	**BD-II**	**HCs**	**Main effect^a^**			**BD-I vs. HCs**			**BD-II vs. HCs**			**BD-I vs. BD-II**		
**EMM (SE)**				***F***	***p***	**Power**	***t***	***P***	**Power**	***t***	***p***	**Power**	***t***	***p***	**Power**
Left inferior temporal gyrus	2.84 (0.02)	2.81 (0.03)	2.94 (0.03)	6.57	**0.027**	0.917	−2.83	**0.013**	0.808	−3.47	**0.003**	0.930	1.00	0.336	0.173
Left rostral middle frontal gyrus	2.34 (0.02)	2.30 (0.02)	2.40 (0.02)	6.20	**0.034**	0.902	−2.30	**0.036**	0.657	−3.45	**0.003**	0.931	1.52	0.159	0.338
Left superior frontal gyrus	2.83 (0.02)	2.77 (0.03)	2.93 (0.03)	6.59	**0.027**	0.919	−2.53	**0.023**	0.712	−3.62	**0.003**	0.930	1.38	0.200	0.272
Left superior temporal gyrus	2.83 (0.02)	2.77 (0.03)	2.96 (0.03)	8.92	**<0.001**	0.977	−2.95	**0.009**	0.847	−4.23	**<0.001**	0.982	1.59	0.150	0.353
Left transverse temporal gyrus	2.52 (0.03)	2.46 (0.04)	2.65 (0.04)	5.81	**0.049**	0.881	−2.44	**0.026**	0.707	−3.36	**0.003**	0.906	1.18	0.264	0.219
Right middle temporal gyrus	2.92 (0.03)	2.91 (0.03)	3.07 (0.04)	7.76	**0.023**	0.955	−3.52	**0.003**	0.944	−3.47	**0.003**	0.918	0.22	0.835	0.054
Right superior temporal gyrus	2.90 (0.02)	2.82 (0.03)	3.01 (0.03)	9.70	**<0.001**	0.985	−2.81	**0.013**	0.810	−4.43	**<0.001**	0.989	1.97	0.071	0.501

*EMM, estimated marginal means; SE, standard error; BD-I, bipolar disorder type I; BD-II, bipolar disorder type II; HCs, healthy subject controls.*

a *Analysis of covariance (ANCOVA).*

b *Two-sample t-test controlling for age and gender.*

*FDR correction was used for main effect comparisons and post hoc comparisons; p-values are presented after FDR correction.*

*Bold values indicate significant differences with p <0.05*.

### Structural Covariance Analysis

**Figure 1:**
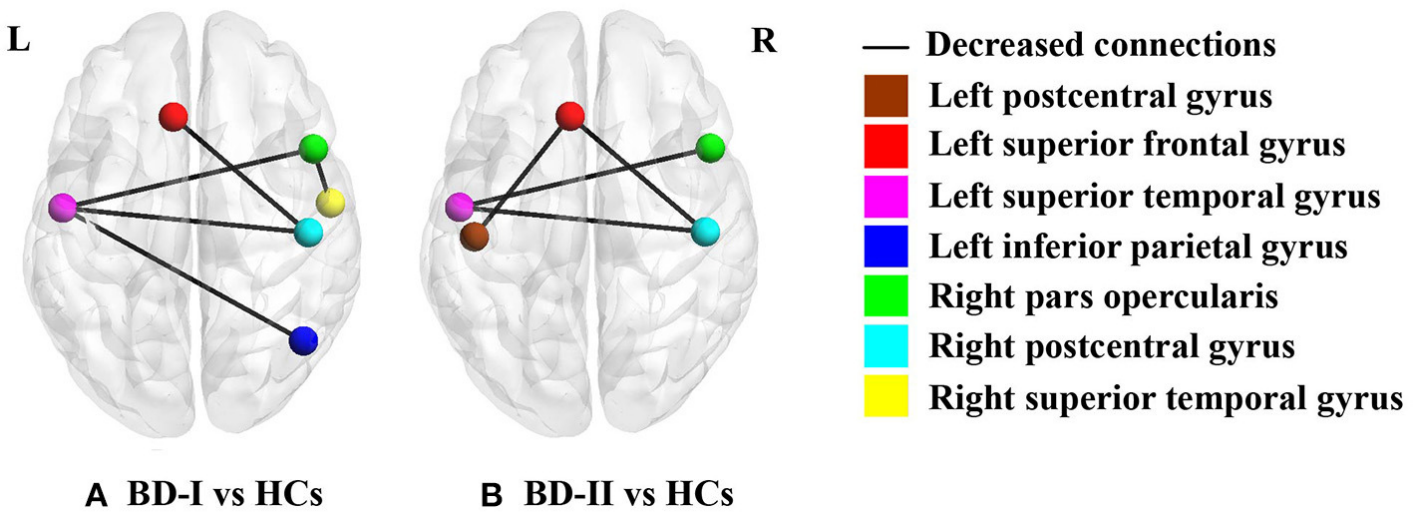
Significant between-group differences of cortical structural covariance. Brain regions are expressed with colored balls, and decreased structural covariance connections between brain regions are represented by solid black lines. **(A)** BD-I group presents significantly decreased structural covariance connections relative to HCs after FDR correction; **(B)** BD-II group shows significantly declined structural covariance connections compared with HCs after FDR correction. BD-I, bipolar disorder type I; BD-II, bipolar disorder type II; HCs, healthy subject controls.

According to the above results of CT analysis, seven cortical regions were regarded as seeds of ROI to analyze SC differences between groups (BD-I vs. HCs, BD-II vs. HCs) (Table 3; Figure 1). Compared with HCs, both BD-I and BD-II groups showed significantly reduced cortical SC connections between left SFG and right postcentral gyrus (PCG), left STG and right pars opercularis, and left STG and right PCG. Apart from these regions, the BD-I group displayed specifically reduced SC connections between left STG and right inferior parietal gyrus (IPG), and right pars opercularis and right STG compared with HCs. Compared with HCs, a significantly decreased cortical SC connection between left PCG and left SFG was only observed in the BD-II group. Interestingly, there was no significant difference in CT between BD-I and BD-II groups, but significant differences of some SC connections were found between BD-I and BD-II groups. The results of all SC analyses are shown in the Supplementary Tables S2, S3 and Supplementary Figure S1.

**Table 3:** Significant between-group differences (BD-I vs. HCs; BD-II vs. HCs) in structural covariance analysis.

**Structural covariance connectivity**	***z***	***p***	**Power**
**BD-I vs. HCs**			
Left superior frontal gyrus and right postcentral gyrus	−3.523	**0.043**	0.941
Left superior temporal gyrus and right inferior parietal	−3.612	**0.034**	0.951
gyrus			
Left superior temporal gyrus and right pars opercularis	−4.709	**0.002**	0.997
Left superior temporal gyrus and right postcentral gyrus	−3.681	**0.034**	0.957
Right pars opercularis and right superior temporal gyrus	−3.907	**0.028**	0.974
**BD-II vs. HCs**			
Left postcentral gyrus and left superior frontal gyrus	−3.708	**0.034**	0.960
Left superior frontal gyrus and right postcentral gyrus	−4.140	**0.016**	0.985
Left superior temporal gyrus and right pars opercularis	−3.616	**0.034**	0.951
Left superior temporal gyrus and right postcentral gyrus	−3.761	**0.034**	0.964

*BD-I, bipolar disorder type I; BD-II, bipolar disorder type II; HCs, healthy subject controls; z, between-group z statistic (BD-I vs. HCs; BD-II vs. HCs).*

*The p-values are presented between-group test after FDR correction for all comparisons.*

*Bold values indicate significant differences with p < 0.05*.

### Cortical Thickness Correlation Analysis

**Figure 2:**
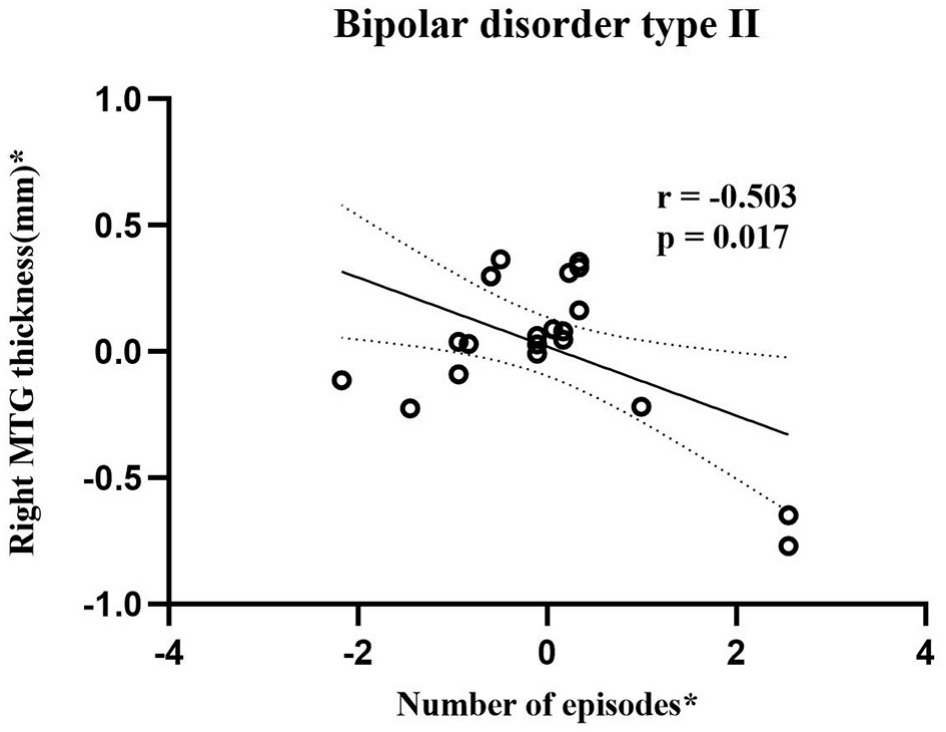
Scatterplots show correlation between cortical thickness in right MTG and number of episodes in adolescent bipolar disorder type II subjects. Asterisk (*) represents the residuals removing the effect of age and gender. The horizontal and vertical axes represent residuals of number of episodes and right MTG thickness, respectively. Bipolar disorder type II group showed significantly negative correlations (*r* = −0.503, *p* = 0.017, Power = 0.695) between cortical thickness in right MTG and number of episodes. MTG stands for middle temporal gyrus.

As shown in Figure 2, BD-II group showed negative correlation (*r* = −0.503, *p* = 0.017, Power = 0.695) between CT in the right MTG and number of episodes.

## Discussion

By calculating CT and cortical SC, the present study probed cortical morphology differences among adolescent BD-I, BD-II, and HCs. The findings of this study were 3-fold: (a) Both BD-I and BD-II groups showed reduced CT in seven cortical regions, including left ITG, left rostral MFG, left SFG, left TTG, right MTG, and bilateral STG. No significant CT difference was found between two BD subtypes. (b) BD-I and BD-II groups shared similarly decreased inter-hemispheric SC connections, including SC connections between left SFG and right PCG, left STG and right pars opercularis, and left STG and right PCG. When comparing with HCs, significantly decreased SC connections between left STG and right IPG and right pars opercularis and right STG were only observed in the BD-I group, and significantly declined SC connection between left PCG with left SFG was only found in the BD-II group. (c) An increase of number of episodes was negatively associated with a thinner right MTG in BD-II patients. Therefore, the discovery of common and specific characteristics in BD-I and BD-II is essential for understanding the neuroanatomy pathophysiology of two BD subtypes though combined CT and cortical SC analysis.

### Common Alteration in CT in BD-I and BD-II

CT is thought to mainly reflect the morphological characteristics of gray matter, such as density, size, and arrangement of cells (38). Our results show that both BD-I and BD-II patients had CT reductions in left rostral MFG and left SFG, which are representative of a functionally defined area DLPFC (39). This result is consistent with a previous neuropathological study, in which declined neuronal and glial density were reported in DLPFC in BD patients (40). Similarly, another review also reported declined density of glia and neurons and decreased size of neurons in frontal areas of BD (41). In addition, in our study, extensive cortical thinning of the temporal gyrus was also found in both BD-I and BD-II patients compared with HCs, including left ITG, left TTG, right MTG, and bilateral STG. Decreased density of neurons in ITG and declined neuronal clustering in the auditory association region in STG and reduced neuronal integrity in TTG were found in BD (42–44). Thus, our findings were in keeping with the neuropathological studies, and decreased density or size of neurons in frontal and temporal regions may be the reasons for its reductions of CT in BD relative to HCs.

These cortical regions showing significant CT differences between groups have different functions. To be specific, rostral MFG and SFG are responsible for generating emotional responses (45) and cognitive control (27), respectively. STG is a critical structure in auditory and language processing and social cognition (46). ITG and MTG participated in high-order visual processing (47), and TTG is involved in auditory information processing (48). Based on previous investigations, the frontal and temporal regions with significant CT differences in the present study were involved in symptoms of emotional (49), cognitive (50), auditory processing (51), and visual impairment (52) in individuals with BD. Moreover, when performing memory, language, cognitive, and affective tasks, BD patients exhibited abnormalities in frontal or temporal regions vs. HCs (53–56). Therefore, we infer that the cortical thinning of several frontal and temporal regions may be associated with memory, emotional, and cognitive dysregulation in adolescent BD-I and BD-II subjects.

In our study, there was no significant difference in CT between BD-I and BD-II groups, possibly because of the small sample size. The result of another study with a relatively small sample size was similar to our finding with no significant difference between two BD subtypes on CT (57). However, a previous study with a large sample size of BD patients found significant differences in CT between BD subtypes. In that study, the CT of right temporal lobe of BD-I patients was significantly lower than that of BD-II patients (8). Lack of significant difference might arise from the small sample size of our study. In future research, we would further study CT with a larger sample size of BD patients. In addition, most BD-I patients had been treated with medication, whereas a relatively small number of BD-II patients had received medication treatment. Previous studies suggest that alterations of brain structure can be normalized in response to medication treatment (58–60). Therefore, medication treatment may also contribute to the finding of no significant difference in CT between BD-I group and BD-II group.

### Common Alteration in SC Connections in BD-I and BD-II

It has been suggested that structural associations are due to a mutual-trophic effect of axonal connection (21). Therefore, changes in axon connections may directly affect cortical morphology, deciding changed patterns of SC (61). In our study, both BD-I and BD-II groups shared decreased SC in left SFG and right PCG, left STG and right pars opercularis of inferior frontal gurus (IFG), and left STG and right PCG. These decreased inter-hemispheric SC connections are similar with results of a diffusion tensor imaging (DTI) study in adult BD. Fractional anisotropy (FA) is a white matter integrity index which reflects axonal coherence (62). Both adult BD-I and BD-II groups showed decreased FA in the corpus callosum relative to HCs, indicating fiber impairments of corpus callosum in both BD subtypes (63). Moreover, adolescent BD with combining BD-I and BD-II subjects were found to show decreased FA in corpus callosum (64). Corpus callosum is a major interhemispheric commissure, which connects most of the neocortical regions (64). Therefore, abnormal changes in axon of corpus callosum in two BD subtype patients may result in the shared decreased inter-hemispheric SC connections in BD-I and BD-II patients.

### Specific Alteration in SC Connections Between BD-I and HCs

In our study, BD-I patients showed a decrease of SC connection between right pars opercularis of IFG and right STG relative to HC. This result is also similar to two previous DTI studies in which declined FA in right uncinate fasciculus was found in BD-I patients but not in BD-II patients (65, 66). Uncinate fasciculus, an intrahemispheric fronto-temporal fiber, connects ventral and orbital parts of the prefrontal cortex and anterior part of temporal gyrus (67). It is a crucial tract of emotion regulation and memory network (68). Thus, this ipsilateral decrease of SC connection may be related to the emotion and memory deficits of BD-I patients. In addition, our study also found a lower SC connection between left STG and right IPG in BD-I subjects relative to HCs. Interestingly, in our study, this decreased inter-hemispheric SC was only in BD-I patients but not in BD-II patients, which was not found in most DTI studies that only found abnormal interhemispheric (decreased FA in corpus callosum) connection in both adult BD-I and BD-II patients. A potential reason may be that the sample in these DTI studies was mostly adult individuals with BD. Besides this, the DTI method is difficult to distinguish the cross fiber and too small tracts at current scanning resolutions (69). Therefore, we infer that SC could be a complimentary method, which provide unique information about the cortico-cortical connection.

### Specific Alteration in SC Connection Between BD-II and HCs

In our study, BD-II patients had a weaker SC connection between left PCG and left SFG vs. HCs. Studies regarding BD-I and BD-II as a whole found that FA in superior longitudinal fasciculus (SLF) was decreased in BD patients relative to HCs (70, 71). SLF, as a major long association fiber tract, ipsilaterally connects parietal, frontal, temporal, and occipital cortices (67). Declined FA in SLF indicated axonal abnormality in BD, which may contribute to our findings of a decrease of cortical SC connection between left PCG and left SFG in BD-II patients. Moreover, prior study indicated that the strength of frontal-parietal correlation was correlated with performance of schizophrenia in recall memory task (72). This study suggests frontal-parietal correlation may be associated with cognitive symptoms. Recent neurobiological research supports that BD and schizophrenia share neurocognitive (73) and genetic characteristics (74), which indicate that two mental diseases are related at the etiological level. Poorer recognition of facial expressions was reported in BD-II patients but not in BD-I patients (75). This cognitive task study results were in accordance with our results. Thus, we infer that the decreased SC connection between left PCG and left SFG may be associated with cognitive impairment in BD-II patients.

### Specific Relationship Between CT and Clinical Variables in BD-II

Correlation analysis results provide evidence that the thickness of right MTG is negatively correlated with the number of episodes in BD-II patients. This finding may indicate that the CT defect in right MTG was more obvious with an increase in the number of episodes in BD-II patients. This result is similar to a longitudinal study focused on adult BD-II patients. This study observed that adult BD-II patients with more depressive number of episodes between baseline and follow-up periods had more severe thinning of the left temporal cortex than patients with fewer depressive numbers of episodes (76). Hence, our finding further supported the association between increased cortical thinning and the number of mood episodes.

## Limitation

In the current study, there are some limitations. First, the sample size of the study is relatively small, especially the number of BD-II patients and HCs. In future work, study needs to be conducted in a broader population of patients and HCs to make the study more convincing. Second, our study is cross-sectional, so we do not know the detailed process of cortical thinning alterations over time in BD patients. Hence, for a better understanding of the cortical changes in BD disease, longitudinal research is needed to help identify the neurodevelopmental process of adolescent BD and understand the procession of abnormalities and the factors that lead to them. Third, because some patients were under medication treatment, medication may have potential influences in our findings. However, influences of medication are unlikely to account for our results as our findings of decreased CT and declined SC connections represent brain structural abnormalities, whereas previous studies prove that changes of brain structure can be normalized in response to medication treatment (58–60). In addition, a previous study found that depressive/manic episodes may have a different effect on brain structure in BD (77). Our study did not collect the type of episodes, so future work needs to further explore the relationship between a different type of number of episodes and cortical thickness.

## Conclusion

In short, we estimate cortical morphology differences among the adolescent BD-I, BD-II, and HCs by calculating CT and cortical SC. Cortical thinning in frontal and temporal cortices was common to adolescent BD-I and BD-II, therefore, which was not sensitive to differences in BD subtype. However, decreased SC connections among frontal, temporal, and parietal regions were common or specific to BD-I and BD-II subjects. The findings of common and specific patterns of cortical morphology alterations may not only be useful for understanding neuroanatomical mechanisms and pathophysiology of two BD subtypes, but also treatment and diagnose of BD-I and BD-II. In addition, the negative correlation between CT in right MTG and number of episodes in BD-II patients suggests that, with an increase of number of episodes, the CT defect of right MTG was more obvious.

## Data Availability Statement

The raw data supporting the conclusions of this article will be made available by the authors, without undue reservation.

## Ethics Statement

The studies involving human participants were reviewed and approved by Ethics Committee of The Second Xiangya Hospital of Central South University. Written informed consent to participate in this study was provided by the participants' legal guardian/next of kin.

## Author Contributions

QJ and GL: conception and study design. WG and LS: magnetic resonance data acquisition and clinical support. ZL, DC, and LK: statistical analysis and data interpretation. YG and JQ: interpretation of results. LK and WC: paper writing and important revision of the article. All authors: approval of final version to be published and agreement to be accountable for the integrity and accuracy of all aspects of the work.

## Funding

The present work was supported by the Funds for the National Natural Science Foundation of China (81371531 to QJ; 81901725 to ZL; and 81901730 to WC), Natural Science Foundation of Zhejiang Province (LQ19H090018 to WG), High-level cultivation program of Shandong First Medical University & Shandong Academy of Medical Sciences (2017GCC11 to WC, Taishan Scholars Program of Shandong Province (TS201712065 to JQ), and Academic Promotion Programme of Shandong First Medical University (2019QL009 to JQ).

## Conflict of Interest

The authors declare that the research was conducted in the absence of any commercial or financial relationships that could be construed as a potential conflict of interest.

## Publisher's Note

All claims expressed in this article are solely those of the authors and do not necessarily represent those of their affiliated organizations, or those of the publisher, the editors and the reviewers. Any product that may be evaluated in this article, or claim that may be made by its manufacturer, is not guaranteed or endorsed by the publisher.

## References

[1] Merikangas KR Jin R He JP Kessler RC Lee S Sampson NA. Prevalence and correlates of bipolar spectrum disorder in the world mental health survey initiative. Arch Gen Psychiatry. (2011) 68:241–51. 10.1001/archgenpsychiatry.2011.12 21383262 PMC3486639

[2] Ekman M Granstrom O Omerov S Jacob J Landen M. The societal cost of bipolar disorder in Sweden. Soc Psychiatry Psychiatr Epidemiol. (2013) 48:1601–10. 10.1007/s00127-013-0724-9 23754681

[3] Palsson E Figueras C Johansson AG Ekman CJ Hultman B Ostlind J. Neurocognitive function in bipolar disorder: a comparison between bipolar I and II disorder and matched controls. BMC Psychiatry. (2013) 13:165. 10.1186/1471-244X-13-165 23758923 PMC3691847

[4] Sagar R Pattanayak RD. Potential biomarkers for bipolar disorder: where do we stand? Indian J Med Res. (2017) 145:7–16. 10.4103/ijmr.IJMR_1386_16 28574009 PMC5460576

[5] Rigucci S Serafini G Pompili M Kotzalidis GD Tatarelli R. Anatomical and functional correlates in major depressive disorder: the contribution of neuroimaging studies. World J Biol Psychiatry. (2010) 11:165–80. 10.3109/15622970903131571 19670087

[6] Wise T Radua J Via E Cardoner N Abe O Adams TM. Common and distinct patterns of grey-matter volume alteration in major depression and bipolar disorder: evidence from voxel-based meta-analysis. Mol Psychiatry. (2017) 22:1455–63. 10.1038/mp.2016.72 27217146 PMC5622121

[7] Kapczinski NS Mwangi B Cassidy RM Librenza-Garcia D Bermudez MB Kauer-Sant'anna M. Neuroprogression and illness trajectories in bipolar disorder. Expert Rev Neurother. (2017) 17:277–85. 10.1080/14737175.2017.1240615 27659841

[8] Abe C Ekman CJ Sellgren C Petrovic P Ingvar M Landen M. Cortical thickness, volume and surface area in patients with bipolar disorder types I and II. J Psychiatry Neurosci. (2016) 41:240–50. 10.1503/jpn.150093 26645741 PMC4915933

[9] Mahon K Wu J Malhotra AK Burdick KE DeRosse P Ardekani BA. A voxel-based diffusion tensor imaging study of white matter in bipolar disorder. Neuropsychopharmacology. (2009) 34:1590–600. 10.1038/npp.2008.216 19145224 PMC2811531

[10] Hanford LC Nazarov A Hall GB Sassi RB. Cortical thickness in bipolar disorder: a systematic review. Bipolar Disord. (2016) 18:4–18. 10.1111/bdi.12362 26851067

[11] Rimol LM Hartberg CB Nesvag R Fennema-Notestine C Hagler DJ Jr. Cortical thickness and subcortical volumes in schizophrenia and bipolar disorder. Biol Psychiatry. (2010) 68:41–50. 10.1016/j.biopsych.2010.03.036 20609836

[12] Hegarty CE Foland-Ross LC Narr KL Sugar CA McGough JJ Thompson PM. ADHD comorbidity can matter when assessing cortical thickness abnormalities in patients with bipolar disorder. Bipolar Disord. (2012) 14:843–55. 10.1111/bdi.12024 23167934 PMC3506177

[13] Janssen J Aleman-Gomez Y Schnack H Balaban E Pina-Camacho L Alfaro-Almagro F. Cortical morphology of adolescents with bipolar disorder and with schizophrenia. Schizophr Res. (2014) 158:91–9. 10.1016/j.schres.2014.06.040 25085384

[14] Niu M Wang Y Jia Y Wang J Zhong S Lin J. Common and specific abnormalities in cortical thickness in patients with major depressive and bipolar disorders. EBioMedicine. (2017) 16:162–71. 10.1016/j.ebiom.2017.01.010 28109831 PMC5474436

[15] Sultan AA Kennedy KG Fiksenbaum L MacIntosh BJ Goldstein BI. Neurostructural correlates of cannabis use in adolescent bipolar disorder. Int J Neuropsychopharmacol. (2021) 24:181–90. 10.1093/ijnp/pyaa077 33103721 PMC7968618

[16] Savitz JB Price JL Drevets WC. Neuropathological and neuromorphometric abnormalities in bipolar disorder: view from the medial prefrontal cortical network. Neurosci Biobehav Rev. (2014) 42:132–47. 10.1016/j.neubiorev.2014.02.008 24603026

[17] Toma S Islam AH Metcalfe AWS Mitchell RHB Fiksenbaum L MacIntosh BJ. Cortical volume and thickness across bipolar disorder subtypes in adolescents: a preliminary study. J Child Adolesc Psychopharmacol. (2019) 29:141–51. 10.1089/cap.2017.0137 30359542

[18] Hibar DP Westlye LT Doan NT Jahanshad N Cheung JW Ching CRK. Cortical abnormalities in bipolar disorder: an MRI analysis of 6503 individuals from the ENIGMA Bipolar Disorder Working Group. Mol Psychiatry. (2018) 23:932–42. 10.1038/mp.2017.73 28461699 PMC5668195

[19] Joshi SH Vizueta N Foland-Ross L Townsend JD Bookheimer SY Thompson PM. Relationships between altered functional magnetic resonance imaging activation and cortical thickness in patients with euthymic bipolar i disorder. Biol Psychiatry Cogn Neurosci Neuroimaging. (2016) 1:507–17. 10.1016/j.bpsc.2016.06.006 27990494 PMC5157843

[20] Alexander-Bloch A Raznahan A Bullmore E Giedd J. The convergence of maturational change and structural covariance in human cortical networks. J Neurosci. (2013) 33:2889–99. 10.1523/JNEUROSCI.3554-12.2013 23407947 PMC3711653

[21] Mechelli A Friston KJ Frackowiak RS Price CJ. Structural covariance in the human cortex. J Neurosci. (2005) 25:8303–10. 10.1523/JNEUROSCI.0357-05.2005 16148238 PMC6725541

[22] Kelly C Toro R Di Martino A Cox CL Bellec P Castellanos FX. A convergent functional architecture of the insula emerges across imaging modalities. Neuroimage. (2012) 61:1129–42. 10.1016/j.neuroimage.2012.03.021 22440648 PMC3376229

[23] Gong G He Y Chen ZJ Evans AC. Convergence and divergence of thickness correlations with diffusion connections across the human cerebral cortex. Neuroimage. (2012) 59:1239–48. 10.1016/j.neuroimage.2011.08.017 21884805

[24] Wannan CMJ Cropley VL Chakravarty MM Bousman C Ganella EP Bruggemann JM. Evidence for network-based cortical thickness reductions in schizophrenia. Am J Psychiatry. (2019) 176:552–63. 10.1176/appi.ajp.2019.18040380 31164006

[25] Caeyenberghs K Taymans T Wilson PH Vanderstraeten G Hosseini H van Waelvelde H. Neural signature of developmental coordination disorder in the structural connectome independent of comorbid autism. Dev Sci. (2016) 19:599–612. 10.1111/desc.12424 27147441

[26] Li X Cao Q Pu F Li D Fan Y An L. Abnormalities of structural covariance networks in drug-naive boys with attention deficit hyperactivity disorder. Psychiatry Res. (2015) 231:273–8. 10.1016/j.pscychresns.2015.01.006 25682468

[27] Kim S Kim YW Jeon H Im CH Lee SH. Altered cortical thickness-based individualized structural covariance networks in patients with schizophrenia and bipolar disorder. J Clin Med. (2020) 9. 10.3390/jcm9061846 32545747 PMC7356298

[28] Han S Cui Q Wang X Chen Y Li D Li L. The anhedonia is differently modulated by structural covariance network of NAc in bipolar disorder and major depressive disorder. Prog Neuropsychopharmacol Biol Psychiatry. (2020) 99:109865. 10.1016/j.pnpbp.2020.109865 31962188

[29] Yeh PH Zhu H Nicoletti MA Hatch JP Brambilla P Soares JC. Structural equation modeling and principal component analysis of gray matter volumes in major depressive and bipolar disorders: differences in latent volumetric structure. Psychiatry Res. (2010) 184:177–85. 10.1016/j.pscychresns.2010.07.007 21051206 PMC3001135

[30] Keshavan MS Giedd J Lau JYF Lewis DA Paus T. Changes in the adolescent brain and the pathophysiology of psychotic disorders. Lancet Psychiatry. (2014) 1:549–58. 10.1016/S2215-0366(14)00081-9 26361314

[31] Kaufman J Birmaher B Brent D Rao U Flynn C Moreci P. Schedule for affective disorders and schizophrenia for school-age children-present and lifetime version (K-SADS-PL): initial reliability and validity data. J Am Acad Child Adolesc Psychiatry. (1997) 36:980–8. 10.1097/00004583-199707000-00021 9204677

[32] Young RC Biggs JT Ziegler VE Meyer DA. A rating scale for mania: reliability, validity and sensitivity. Br J Psychiatry. (1978) 133:429–35. 10.1192/bjp.133.5.429 728692

[33] Wood A Kroll L Moore A Harrington R. Properties of the mood and feelings questionnaire in adolescent psychiatric outpatients: a research note. J Child Psychol Psychiatry. (1995) 36:327–34. 10.1111/j.1469-7610.1995.tb01828.x 7759594

[34] Desikan RS Segonne F Fischl B Quinn BT Dickerson BC Blacker D. An automated labeling system for subdividing the human cerebral cortex on MRI scans into gyral based regions of interest. Neuroimage. (2006) 31:968–80. 10.1016/j.neuroimage.2006.01.021 16530430

[35] Benjamini Y Hochberg Y. Controlling the false discovery rate: a practical and powerful approach to multiple testing. J R Stat Soc Ser B Methodol. (1995) 57:289–300. 10.1111/j.2517-6161.1995.tb02031.x

[36] Ashburner J Csernansk JG Davatzikos C Fox NC Frisoni GB Thompson PM. Computer-assisted imaging to assess brain structure in healthy and diseased brains. Lancet Neurol. (2003) 2:79–88. 10.1016/S1474-4422(03)00304-1 12849264

[37] Duan X Wang R Xiao J Li Y Huang X Guo X. Subcortical structural covariance in young children with autism spectrum disorder. Prog Neuropsychopharmacol Biol Psychiatry. (2020) 99:109874. 10.1016/j.pnpbp.2020.109874 31981719

[38] Kuang C Buchy L Barbato M Makowski C MacMaster FP Bray S. A pilot study of cognitive insight and structural covariance in first-episode psychosis. Schizophr Res. (2017) 179:91–6. 10.1016/j.schres.2016.09.036 27720314

[39] Zhang L Opmeer EM van der Meer L Aleman A Curcic-Blake B Ruhe HG. Altered frontal-amygdala effective connectivity during effortful emotion regulation in bipolar disorder. Bipolar Disord. (2018) 20:349–58. 10.1111/bdi.12611 29430790

[40] Rajkowska G Halaris A Selemon LD. Reductions in neuronal and glial density characterize the dorsolateral prefrontal cortex in bipolar disorder. Biol Psychiatry. (2001) 49:741–52. 10.1016/S0006-3223(01)01080-0 11331082

[41] Gigante AD Young LT Yatham LN Andreazza AC Nery FG Grinberg LT. Morphometric post-mortem studies in bipolar disorder: possible association with oxidative stress and apoptosis. Int J Neuropsychopharmacol. (2011) 14:1075–89. 10.1017/S146114571000146X 21205433

[42] Yoshikawa T Hayashi Y Nihonmatsu-Kikuchi N Hisanaga S-i Yu X-j Tatebayashi Y. Neuropathological similarities and differences between schizophrenia and bipolar disorder: a flow cytometric postmortem brain study. PLoS ONE. (2012) 7. 10.1371/journal.pone.0033019 22438888 PMC3305297

[43] Beasley CL Chana G Honavar M Landau S Everall IP Cotter D. Evidence for altered neuronal organisation within the planum temporale in major psychiatric disorders. Schizophr Res. (2005) 73:69–78. 10.1016/j.schres.2004.08.011 15567079

[44] Nudmamud S Reynolds LM Reynolds GP. N-acetylaspartate and N-Acetylaspartylglutamate deficits in superior temporal cortex in schizophrenia and bipolar disorder: a postmortem study. Biol Psychiatry. (2003) 53:1138–41. 10.1016/S0006-3223(02)01742-0 12814865

[45] Etkin A Egner T Kalisch R. Emotional processing in anterior cingulate and medial prefrontal cortex. Trends Cogn Sci. (2011) 15:85–93. 10.1016/j.tics.2010.11.004 21167765 PMC3035157

[46] Bigler ED Mortensen S Neeley ES Ozonoff S Krasny L Johnson M. Superior temporal gyrus, language function, and autism. Dev Neuropsychol. (2007) 31:217–38. 10.1080/87565640701190841 17488217

[47] Sewards TV Sewards MA. On the neural correlates of object recognition awareness: relationship to computational activities and activities mediating perceptual awareness. Conscious Cogn. (2002) 11:51–77. 10.1006/ccog.2001.0518 11883988

[48] Warrier C Wong P Penhune V Zatorre R Parrish T Abrams D. Relating structure to function: Heschl's gyrus and acoustic processing. J Neurosci. (2009) 29:61–9. 10.1523/JNEUROSCI.3489-08.2009 19129385 PMC3341414

[49] Isik Ulusoy S Gulseren SA Ozkan N Bilen C. Facial emotion recognition deficits in patients with bipolar disorder and their healthy parents. Gen Hosp Psychiatry. (2020) 65:9–14. 10.1016/j.genhosppsych.2020.04.008 32361661

[50] Lima IMM Peckham AD Johnson SL. Cognitive deficits in bipolar disorders: implications for emotion. Clin Psychol Rev. (2018) 59:126–36. 10.1016/j.cpr.2017.11.006 29195773 PMC6404979

[51] Zenisek R Thaler NS Sutton GP Ringdahl EN Snyder JS Allen DN. Auditory processing deficits in bipolar disorder with and without a history of psychotic features. Bipolar Disord. (2015) 17:769–80. 10.1111/bdi.12333 26396062

[52] Fernandes TP Silverstein SM Almeida NL Santos NA. Visual impairments in type 1 bipolar disorder. World J Biol Psychiatry. (2019) 20:790–8. 10.1080/15622975.2019.1628302 31169048

[53] Welander-Vatn A Jensen J Otnaess MK Agartz I Server A Melle I. The neural correlates of cognitive control in bipolar I disorder: an fMRI study of medial frontal cortex activation during a Go/No-go task. Neurosci Lett. (2013) 549:51–6. 10.1016/j.neulet.2013.06.010 23778236

[54] Frangou S Dakhil N Landau S Kumari V. Fronto-temporal function may distinguish bipolar disorder from schizophrenia. Bipolar Disord. (2006) 8:47–55. 10.1111/j.1399-5618.2006.00274.x 16411980

[55] Chakrabarty T Kozicky JM Torres IJ Lam RW Yatham LN. Verbal memory impairment in new onset bipolar disorder: relationship with frontal and medial temporal morphology. World J Biol Psychiatry. (2015) 16:249–60. 10.3109/15622975.2014.1000373 25708742

[56] Sankar A Purves K Colic L Cox Lippard ET Millard H Fan S. Altered frontal cortex functioning in emotion regulation and hopelessness in bipolar disorder. Bipolar Disord. (2021) 23:152–64. 10.1111/bdi.12954 32521570 PMC7790437

[57] Woo Y Kang W Kang Y Kim A Han KM Tae WS. Cortical thickness and surface area abnormalities in bipolar I and II disorders. Psychiatry Investig. (2021) 18:850–63. 10.30773/pi.2021.0074 34500506 PMC8473857

[58] Sassi RB Brambilla P Hatch JP Nicoletti MA Mallinger AG Frank E. Reduced left anterior cingulate volumes in untreated bipolar patients. Biol Psychiatry. (2004) 56:467–75. 10.1016/j.biopsych.2004.07.005 15450781

[59] Haznedar MM Roversi F Pallanti S Baldini-Rossi N Schnur DB Licalzi EM. Fronto-thalamo-striatal gray and white matter volumes and anisotropy of their connections in bipolar spectrum illnesses. Biol Psychiatry. (2005) 57:733–42. 10.1016/j.biopsych.2005.01.002 15820230

[60] Bearden CE Thompson PM Dutton RA Frey BN Peluso MA Nicoletti M. Three-dimensional mapping of hippocampal anatomy in unmedicated and lithium-treated patients with bipolar disorder. Neuropsychopharmacology. (2008) 33:1229–38. 10.1038/sj.npp.1301507 17687266 PMC6693586

[61] Hoffman RE Dobscha SK. Cortical pruning and the development of schizophrenia: a computer model. Schizophr Bull. (1989) 15:477–90. 10.1093/schbul/15.3.477 2814376

[62] Ambrosi E Chiapponi C Sani G Manfredi G Piras F Caltagirone C. White matter microstructural characteristics in bipolar I and bipolar II disorder: a diffusion tensor imaging study. J Affect Disord. (2016) 189:176–83. 10.1016/j.jad.2015.09.035 26437232

[63] Ha TH Her JY Kim JH Chang JS Cho HS Ha K. Similarities and differences of white matter connectivity and water diffusivity in bipolar I and II disorder. Neurosci Lett. (2011) 505:150–4. 10.1016/j.neulet.2011.10.009 22008503

[64] Barnea-Goraly N Chang KD Karchemskiy A Howe ME Reiss AL. Limbic and corpus callosum aberrations in adolescents with bipolar disorder: a tract-based spatial statistics analysis. Biol Psychiatry. (2009) 66:238–44. 10.1016/j.biopsych.2009.02.025 19389661

[65] Foley SF Bracher-Smith M Tansey KE Harrison JR Parker GD Caseras X. Fractional anisotropy of the uncinate fasciculus and cingulum in bipolar disorder type I, type II, unaffected siblings and healthy controls. Br J Psychiatry. (2018) 213:548–54. 10.1192/bjp.2018.101 30113288 PMC6130806

[66] Caseras X Murphy K Lawrence NS Fuentes-Claramonte P Watts J Jones DK. Emotion regulation deficits in euthymic bipolar I versus bipolar II disorder: a functional and diffusion-tensor imaging study. Bipolar Disord. (2015) 17:461–70. 10.1111/bdi.12292 25771686 PMC4672703

[67] Catani M Howard RJ Pajevic S Jones DK. Virtual *in vivo* interactive dissection of white matter fasciculi in the human brain. NeuroImage. (2002) 17:77–94. 10.1006/nimg.2002.1136 12482069

[68] Lin F Weng S Xie B Wu G Lei H. Abnormal frontal cortex white matter connections in bipolar disorder: a DTI tractography study. J Affect Disord. (2011) 131:299–306. 10.1016/j.jad.2010.12.018 21236494

[69] Jones DK Knosche TR Turner R. White matter integrity, fiber count, and other fallacies: the do's and don'ts of diffusion MRI. Neuroimage. (2013) 73:239–54. 10.1016/j.neuroimage.2012.06.081 22846632

[70] Lee DK Lee H Park K Joh E Kim CE Ryu S. Common gray and white matter abnormalities in schizophrenia and bipolar disorder. PLoS ONE. (2020) 15:e0232826. 10.1371/journal.pone.0232826 32379845 PMC7205291

[71] Lan MJ Rubin-Falcone H Sublette ME Oquendo MA Stewart JW Hellerstein DJ. Deficits of white matter axial diffusivity in bipolar disorder relative to major depressive disorder: no relationship to cerebral perfusion or body mass index. Bipolar Disord. (2020) 22:296–302. 10.1111/bdi.12845 31604361

[72] Abbs B Liang L Makris N Tsuang M Seidman LJ Goldstein JM. Covariance modeling of MRI brain volumes in memory circuitry in schizophrenia: sex differences are critical. Neuroimage. (2011) 56:1865–74. 10.1016/j.neuroimage.2011.03.079 21497198 PMC3113542

[73] Kim MD Seo HJ Yun HJ Jung YE Park JH Lee CI. The relationship between cognitive decline and psychopathology in patients with schizophrenia and bipolar disorder. Clin Psychopharmacol Neurosci. (2015) 13:103–8. 10.9758/cpn.2015.13.1.103 25912543 PMC4423162

[74] International Schizophrenia C Purcell SM Wray NR Stone JL Visscher PM O'Donovan MC. Common polygenic variation contributes to risk of schizophrenia and bipolar disorder. Nature. (2009) 460:748–52. 10.1038/nature08185 19571811 PMC3912837

[75] Jensen MB Kjaerstad HL Coello K Stanislaus S Melbye S Sletved KO. Affective and non-affective cognition in patients with bipolar disorder type I and type II in full or partial remission: associations with familial risk. J Affect Disord. (2021) 283:207–15. 10.1016/j.jad.2021.01.074 33561801

[76] Zak N Boen E Boye B Andreassen OA Doan NT Malt UF. Mood episodes are associated with increased cortical thinning: a longitudinal study of bipolar disorder type II. Bipolar Disord. (2019) 21:525–38. 10.1111/bdi.12771 30864260

[77] Janiri D Simonetti A Piras F Ciullo V Spalletta G Sani G. Predominant polarity and hippocampal subfield volumes in bipolar disorders. Bipolar Disord. (2020) 22:490–7. 10.1111/bdi.12857 31630469

