# Supplementary material for: Common and Specific Characteristics of Adolescent Bipolar Disorder Types I and II: A Combined Cortical Thickness and Structural Covariance Analysis
Source: Front Psychiatry. 2022 Jan 21;12:750798. doi: 10.3389/fpsyt.2021.750798 (PMC8814452; doi:10.3389/fpsyt.2021.750798)
Supplement: Supplementary file 1 [file Data_Sheet_1.docx]

***Supplemental material***

**Table S1.** Cortical thickness differences among BD-I, BD-II and HCs.

|  |  |  |  |  | |  |  |  |  | Post hoc comparisons ^b^ | | | | | | | |  |
| --- | --- | --- | --- | --- | --- | --- | --- | --- | --- | --- | --- | --- | --- | --- | --- | --- | --- | --- |
| Brain region thickness | BD-I | BD-II | HCs | Main effect ^a^ | | |  | BD-I vs HCs | | |  | BD-II vs HCs | | |  | BD-I vs BD-II | | |
| EMM (SE) |  |  |  | F | P | Power |  | T | P | Power |  | T | P | Power |  | T | P | Power |
| Left banks of superior temporal sulcus | 2.47 (0.03) | 2.39 (0.04) | 2.56 (0.05) | 4.15 | 0.088 | 0.744 |  | - | - | - |  | - | - | - |  | - | - | - |
| Left caudal anterior cingulate | 2.83 (0.04) | 2.76 (0.05) | 2.85 (0.06) | 0.85 | 0.640 | 0.201 |  | - | - | - |  | - | - | - |  | - | - | - |
| Left caudal middle frontal | 2.64 (0.02) | 2.59 (0.03) | 2.68 (0.03) | 3.07 | 0.164 | 0.609 |  | - | - | - |  | - | - | - |  | - | - | - |
| Left cuneus | 1.96 (0.03) | 2.03 (0.04) | 2.01 (0.04) | 0.96 | 0.604 | 0.223 |  | - | - | - |  | - | - | - |  | - | - | - |
| Left entorhinal | 3.28 (0.06) | 3.36 (0.08) | 3.25 (0.08) | 0.63 | 0.730 | 0.158 |  | - | - | - |  | - | - | - |  | - | - | - |
| Left fusiform | 2.80 (0.02) | 2.77 (0.03) | 2.87 (0.03) | 4.31 | 0.088 | 0.763 |  | - | - | - |  | - | - | - |  | - | - | - |
| Left inferior parietal | 2.47 (0.02) | 2.44 (0.03) | 2.54 (0.03) | 2.99 | 0.164 | 0.596 |  | - | - | - |  | - | - | - |  | - | - | - |
| Left inferior temporal | 2.84 (0.02) | 2.81 (0.03) | 2.94 (0.03) | 6.57 | **0.027** | 0.917 |  | -2.83 | **0.013** | 0.808 |  | -3.47 | **0.003** | 0.930 |  | 1.00 | 0.336 | 0.173 |
| Left isthmus cingulate | 2.49 (0.03) | 2.43 (0.04) | 2.55 (0.04) | 2.08 | 0.317 | 0.442 |  | - | - | - |  | - | - | - |  | - | - | - |
| Left lateral occipital | 2.18 (0.02) | 2.24 (0.03) | 2.27 (0.03) | 2.38 | 0.252 | 0.494 |  | - | - | - |  | - | - | - |  | - | - | - |
| Left lateral orbitofrontal | 2.71 (0.02) | 2.70 (0.03) | 2.78 (0.03) | 2.45 | 0.243 | 0.508 |  | - | - | - |  | - | - | - |  | - | - | - |
| Left lingual | 2.08 (0.02) | 2.09 (0.03) | 2.15 (0.03) | 1.67 | 0.396 | 0.360 |  | - | - | - |  | - | - | - |  | - | - | - |
| Left medial orbitofrontal | 2.57 (0.02) | 2.58 (0.03) | 2.59 (0.03) | 0.08 | 0.978 | 0.061 |  | - | - | - |  | - | - | - |  | - | - | - |
| Left middle temporal | 2.84 (0.03) | 2.84 (0.03) | 2.94 (0.03) | 2.97 | 0.164 | 0.590 |  | - | - | - |  | - | - | - |  | - | - | - |
| Left parahippocampal | 2.76 (0.05) | 2.75 (0.06) | 2.75 (0.06) | 0.02 | 0.978 | 0.056 |  | - | - | - |  | - | - | - |  | - | - | - |
| Left paracentral | 2.60(0.02) | 2.55 (0.03) | 2.62 (0.03) | 1.62 | 0.400 | 0.351 |  | - | - | - |  | - | - | - |  | - | - | - |
| Left pars opercularis | 2.61 (0.03) | 2.61 (0.03) | 2.71 (0.04) | 3.16 | 0.155 | 0.622 |  | - | - | - |  | - | - | - |  | - | - | - |
| Left pars orbitalis | 2.66 (0.03) | 2.65 (0.04) | 2.67 (0.04) | 0.04 | 0.978 | 0.056 |  | - | - | - |  | - | - | - |  | - | - | - |
| Left pars triangularis | 2.45 (0.03) | 2.45 (0.03) | 2.48 (0.04) | 0.30 | 0.855 | 0.098 |  | - | - | - |  | - | - | - |  | - | - | - |
| Left pericalcarine | 1.69 (0.03) | 1.72 (0.03) | 1.69 (0.04) | 0.42 | 0.816 | 0.124 |  | - | - | - |  | - | - | - |  | - | - | - |
| Left postcentral | 2.08 (0.03) | 2.08 (0.03) | 2.16 (0.04) | 2.01 | 0.322 | 0.428 |  | - | - | - |  | - | - | - |  | - | - | - |
| Left posterior cingulate | 2.68 (0.02) | 2.68 (0.03) | 2.73 (0.03) | 0.95 | 0.604 | 0.223 |  | - | - | - |  | - | - | - |  | - | - | - |
| Left precentral | 2.63 (0.02) | 2.60 (0.03) | 2.72 (0.03) | 4.10 | 0.088 | 0.739 |  | - | - | - |  | - | - | - |  | - | - | - |
| Left precuneus | 2.50 (0.02) | 2.49 (0.02) | 2.58 (0.03) | 5.18 | 0.060 | 0.840 |  | - | - | - |  | - | - | - |  | - | - | - |
| Left rostral anterior cingulate | 2.99 (0.04) | 2.94 (0.05) | 2.95 (0.05) | 0.40 | 0.816 | 0.117 |  | - | - | - |  | - | - | - |  | - | - | - |
| Left rostral middle frontal | 2.34 (0.02) | 2.30 (0.02) | 2.40 (0.02) | 6.20 | **0.034** | 0.902 |  | -2.30 | **0.036** | 0.657 |  | -3.45 | **0.003** | 0.931 |  | 1.52 | 0.159 | 0.338 |
| Left superior frontal | 2.83 (0.02) | 2.77 (0.03) | 2.93 (0.03) | 6.59 | **0.027** | 0.919 |  | -2.53 | **0.023** | 0.712 |  | -3.62 | **0.003** | 0.930 |  | 1.38 | 0.200 | 0.272 |
| Left superior parietal | 2.23 (0.02) | 2.22 (0.03) | 2.31 (0.03) | 3.32 | 0.150 | 0.646 |  | - | - | - |  | - | - | - |  | - | - | - |
| Left superior temporal | 2.83 (0.02) | 2.77 (0.03) | 2.96 (0.03) | 8.92 | **<0.001** | 0.977 |  | -2.95 | **0.009** | 0.847 |  | -4.23 | **<0.001** | 0.982 |  | 1.59 | 0.150 | 0.353 |
| Left supramarginal | 2.57 (0.02) | 2.52 (0.03) | 2.63 (0.03) | 4.05 | 0.088 | 0.734 |  | - | - | - |  | - | - | - |  | - | - | - |
| Left frontal pole | 2.74 (0.03) | 2.75 (0.04) | 2.82 (0.04) | 1.13 | 0.544 | 0.260 |  | - | - | - |  | - | - | - |  | - | - | - |
| Left temporal pole | 3.56 (0.05) | 3.59 (0.06) | 3.59 (0.07) | 0.06 | 0.978 | 0.061 |  | - | - | - |  | - | - | - |  | - | - | - |
| Left transverse temporal | 2.52 (0.03) | 2.46 (0.04) | 2.65 (0.04) | 5.81 | **0.049** | 0.881 |  | -2.44 | **0.026** | 0.707 |  | -3.36 | **0.003** | 0.906 |  | 1.18 | 0.264 | 0.219 |
| Left insula | 3.13 (0.02) | 3.13 (0.02) | 3.16 (0.03) | 0.49 | 0.789 | 0.130 |  | - | - | - |  | - | - | - |  | - | - | - |
| Right banks of superior temporal sulcus | 2.65 (0.03) | 2.59 (0.04) | 2.74 (0.04) | 3.39 | 0.147 | 0.652 |  | - | - | - |  | - | - | - |  | - | - | - |
| Right caudal anterior cingulate | 2.68 (0.04) | 2.63 (0.05) | 2.57 (0.05) | 1.26 | 0.493 | 0.283 |  | - | - | - |  | - | - | - |  | - | - | - |
| Right caudal middle frontal | 2.59 (0.03) | 2.59 (0.03) | 2.69 (0.03) | 2.87 | 0.171 | 0.577 |  | - | - | - |  | - | - | - |  | - | - | - |
| Right cuneus | 1.99 (0.03) | 1.99 (0.03) | 2.07 (0.03) | 1.80 | 0.368 | 0.390 |  | - | - | - |  | - | - | - |  | - | - | - |
| Right entorhinal | 3.49 (0.07) | 3.57 (0.08) | 3.43 (0.09) | 0.64 | 0.730 | 0.158 |  | - | - | - |  | - | - | - |  | - | - | - |
| Right fusiform | 2.86 (0.02) | 2.88 (0.02) | 2.92 (0.03) | 1.52 | 0.412 | 0.329 |  | - | - | - |  | - | - | - |  | - | - | - |
| Right inferior parietal | 2.54 (0.02) | 2.50 (0.03) | 2.56 (0.03) | 1.52 | 0.412 | 0.337 |  | - | - | - |  | - | - | - |  | - | - | - |
| Right inferior temporal | 2.98 (0.02) | 2.94 (0.03) | 3.05 (0.03) | 3.18 | 0.155 | 0.622 |  | - | - | - |  | - | - | - |  | - | - | - |
| Right isthmus cingulate | 2.46 (0.03) | 2.49 (0.04) | 2.50 (0.04) | 0.40 | 0.816 | 0.117 |  | - | - | - |  | - | - | - |  | - | - | - |
| Right lateral occipital | 2.31 (0.02) | 2.32 (0.03) | 2.38 (0.03) | 1.87 | 0.355 | 0.398 |  | - | - | - |  | - | - | - |  | - | - | - |
| Right lateral orbitofrontal | 2.71 (0.02) | 2.71 (0.03) | 2.74 (0.03) | 0.36 | 0.835 | 0.110 |  | - | - | - |  | - | - | - |  | - | - | - |
| Right lingual | 2.16 (0.03) | 2.19 (0.03) | 2.17 (0.03) | 0.28 | 0.858 | 0.098 |  | - | - | - |  | - | - | - |  | - | - | - |
| Right medial orbitofrontal | 2.50 (0.02) | 2.54 (0.03) | 2.53 (0.03) | 0.64 | 0.730 | 0.158 |  | - | - | - |  | - | - | - |  | - | - | - |
| Right middle temporal | 2.92 (0.03) | 2.91 (0.03) | 3.07 (0.04) | 7.76 | **0.023** | 0.955 |  | -3.52 | **0.003** | 0.944 |  | -3.47 | **0.003** | 0.918 |  | 0.22 | 0.835 | 0.054 |
| Right parahippocampal | 2.74 (0.04) | 2.72 (0.06) | 2.71 (0.06) | 0.08 | 0.978 | 0.061 |  | - | - | - |  | - | - | - |  | - | - | - |
| Right paracentral | 2.58 (0.02) | 2.55 (0.03) | 2.68 (0.03) | 5.20 | 0.060 | 0.840 |  | - | - | - |  | - | - | - |  | - | - | - |
| Right pars opercularis | 2.68 (0.03) | 2.68 (0.04) | 2.74 (0.04) | 0.92 | 0.610 | 0.215 |  | - | - | - |  | - | - | - |  | - | - | - |
| Right pars orbitalis | 2.71 (0.03) | 2.68 (0.04) | 2.75 (0.04) | 0.81 | 0.651 | 0.193 |  | - | - | - |  | - | - | - |  | - | - | - |
| Right pars triangularis | 2.51 (0.03) | 2.53 (0.03) | 2.56 (0.04) | 0.58 | 0.751 | 0.151 |  | - | - | - |  | - | - | - |  | - | - | - |
| Right pericalcarine | 1.70 (0.03) | 1.72 (0.04) | 1.68 (0.04) | 0.26 | 0.863 | 0.091 |  | - | - | - |  | - | - | - |  | - | - | - |
| Right postcentral | 2.09 (0.03) | 2.12 (0.03) | 2.17 (0.04) | 1.66 | 0.396 | 0.360 |  | - | - | - |  | - | - | - |  | - | - | - |
| Right posterior cingulate | 2.61 (0.02) | 2.61 (0.03) | 2.63 (0.03) | 0.14 | 0.953 | 0.073 |  | - | - | - |  | - | - | - |  | - | - | - |
| Right precentral | 2.60 (0.02) | 2.60 (0.03) | 2.70 (0.03) | 4.62 | 0.080 | 0.794 |  | - | - | - |  | - | - | - |  | - | - | - |
| Right precuneus | 2.48 (0.02) | 2.47 (0.03) | 2.57 (0.03) | 4.61 | 0.080 | 0.790 |  | - | - | - |  | - | - | - |  | - | - | - |
| Right rostral anterior cingulate | 3.02 (0.04) | 2.93 (0.05) | 2.91 (0.06) | 1.46 | 0.417 | 0.321 |  | - | - | - |  | - | - | - |  | - | - | - |
| Right rostral middle frontal | 2.32 (0.02) | 2.31 (0.02) | 2.35 (0.02) | 1.08 | 0.559 | 0.245 |  | - | - | - |  | - | - | - |  | - | - | - |
| Right superior frontal | 2.75 (0.02) | 2.72 (0.03) | 2.82 (0.03) | 4.13 | 0.088 | 0.744 |  | - | - | - |  | - | - | - |  | - | - | - |
| Right superior parietal | 2.24 (0.02) | 2.23 (0.03) | 2.29 (0.03) | 1.50 | 0.412 | 0.329 |  | - | - | - |  | - | - | - |  | - | - | - |
| Right superior temporal | 2.90(0.02) | 2.82 (0.03) | 3.01 (0.03) | 9.70 | **<0.001** | 0.985 |  | -2.81 | **0.013** | 0.810 |  | -4.43 | **<0.001** | 0.989 |  | 1.97 | 0.071 | 0.501 |
| Right supramarginal | 2.63 (0.02) | 2.58 (0.03) | 2.67 (0.03) | 2.06 | 0.317 | 0.435 |  | - | - | - |  | - | - | - |  | - | - | - |
| Right frontal pole | 2.75 (0.06) | 2.82 (0.07) | 2.76 (0.08) | 0.32 | 0.851 | 0.104 |  | - | - | - |  | - | - | - |  | - | - | - |
| Right temporal pole | 3.67 (0.05) | 3.65 (0.06) | 3.67 (0.07) | 0.03 | 0.978 | 0.056 |  | - | - | - |  | - | - | - |  | - | - | - |
| Right transverse temporal | 2.49 (0.04) | 2.43 (0.05) | 2.62 (0.05) | 4.25 | 0.088 | 0.758 |  | - | - | - |  | - | - | - |  | - | - | - |
| Right insula | 3.14 (0.03) | 3.17 (0.04) | 3.19 (0.04) | 0.53 | 0.774 | 0.749 |  | - | - | - |  | - | - | - |  | - | - | - |

EMM, estimated marginal means; SE, standard error; BD-I, bipolar disorder type I; BD-II, bipolar disorder type II; HCs, healthy subject controls.^a^Analysis of covariance (ANCOVA) and ^b^Two-sample t-test controlling for age and gender.FDR correction for main effect comparisons and post hoc comparisons; P values presented after FDR correction.Bold values indicating significant differences with p < 0.05.

**Table S2.** Structural covariance differences of BD-I vs HCs.

| Structural covariance connectivity | Left inferior temporal | | |  | Left rostral middle frontal | | |  | Left superior frontal | | |  | Left superior temporal | | |  | Left transverse temporal | | |  | Right middle temporal | | |  | Right superior temporal | | |
| --- | --- | --- | --- | --- | --- | --- | --- | --- | --- | --- | --- | --- | --- | --- | --- | --- | --- | --- | --- | --- | --- | --- | --- | --- | --- | --- | --- |
|  | z | p | Power |  | z | p | Power |  | z | p | Power |  | z | p | Power |  | z | p | Power |  | z | p | Power |  | z | p | Power |
| Left banks of superior temporal sulcus | -0.334 | 0.890 | 0.178 |  | 0.442 | 0.850 | 0.073 |  | 0.385 | 0.872 | 0.067 |  | 0.268 | 0.915 | 0.058 |  | -0.034 | 0.995 | 0.050 |  | -1.054 | 0.620 | 0.184 |  | 0.985 | 0.652 | 0.167 |
| Left caudal anterior cingulate | -0.594 | 0.790 | 0.063 |  | -1.484 | 0.447 | 0.317 |  | -0.774 | 0.727 | 0.121 |  | -1.314 | 0.512 | 0.260 |  | -1.376 | 0.486 | 0.280 |  | -0.785 | 0.727 | 0.123 |  | -0.331 | 0.891 | 0.063 |
| Left caudal middle frontal | -0.494 | 0.832 | 0.125 |  | 0.343 | 0.885 | 0.064 |  | -1.458 | 0.457 | 0.308 |  | -2.161 | 0.235 | 0.580 |  | -0.774 | 0.727 | 0.121 |  | 0.442 | 0.850 | 0.073 |  | -1.618 | 0.394 | 0.366 |
| Left cuneus | -0.711 | 0.744 | 0.233 |  | -0.921 | 0.679 | 0.151 |  | -0.428 | 0.856 | 0.071 |  | -0.005 | 0.996 | 0.050 |  | -1.637 | 0.388 | 0.373 |  | -0.599 | 0.789 | 0.092 |  | -0.319 | 0.898 | 0.062 |
| Left entorhinal | -0.716 | 0.744 | 0.301 |  | 1.100 | 0.600 | 0.196 |  | 0.452 | 0.850 | 0.074 |  | 0.153 | 0.957 | 0.053 |  | 0.297 | 0.911 | 0.060 |  | -0.043 | 0.992 | 0.050 |  | 0.014 | 0.996 | 0.050 |
| Left fusiform | -1.397 | 0.477 | 0.526 |  | 1.510 | 0.437 | 0.326 |  | 0.074 | 0.979 | 0.051 |  | -0.458 | 0.850 | 0.074 |  | -0.338 | 0.888 | 0.063 |  | -3.125 | 0.070 | 0.878 |  | -1.120 | 0.593 | 0.202 |
| Left inferior parietal | -0.630 | 0.774 | 0.102 |  | 0.106 | 0.969 | 0.051 |  | -1.637 | 0.388 | 0.373 |  | -2.733 | 0.121 | 0.780 |  | -1.745 | 0.362 | 0.415 |  | -0.509 | 0.830 | 0.080 |  | -1.545 | 0.424 | 0.339 |
| Left inferior temporal | - | - | - |  | -0.480 | 0.839 | 0.077 |  | -0.584 | 0.796 | 0.090 |  | -0.432 | 0.855 | 0.072 |  | -1.393 | 0.479 | 0.286 |  | -1.365 | 0.493 | 0.276 |  | 0.047 | 0.990 | 0.050 |
| Left isthmus cingulate | -0.443 | 0.850 | 0.075 |  | -0.571 | 0.802 | 0.088 |  | -0.675 | 0.761 | 0.104 |  | 0.644 | 0.767 | 0.099 |  | -1.317 | 0.512 | 0.261 |  | -0.402 | 0.865 | 0.069 |  | -0.240 | 0.923 | 0.057 |
| Left lateral occipital | -1.111 | 0.596 | 0.136 |  | -0.374 | 0.874 | 0.066 |  | -0.880 | 0.689 | 0.142 |  | -2.613 | 0.134 | 0.743 |  | -1.841 | 0.342 | 0.453 |  | -0.264 | 0.916 | 0.058 |  | -2.177 | 0.228 | 0.586 |
| Left lateral orbitofrontal | 0.006 | 0.996 | 0.052 |  | -1.411 | 0.475 | 0.292 |  | -0.403 | 0.865 | 0.069 |  | -0.631 | 0.774 | 0.097 |  | -1.466 | 0.457 | 0.311 |  | 0.689 | 0.757 | 0.106 |  | -0.065 | 0.982 | 0.050 |
| Left lingual | -0.891 | 0.689 | 0.115 |  | -0.374 | 0.874 | 0.066 |  | -0.931 | 0.677 | 0.154 |  | -0.301 | 0.909 | 0.060 |  | -0.160 | 0.955 | 0.053 |  | -1.064 | 0.618 | 0.186 |  | -1.341 | 0.504 | 0.268 |
| Left medial orbitofrontal | -0.438 | 0.852 | 0.457 |  | 0.123 | 0.963 | 0.052 |  | -0.578 | 0.798 | 0.089 |  | -0.504 | 0.831 | 0.080 |  | -0.226 | 0.929 | 0.056 |  | 0.021 | 0.996 | 0.050 |  | 0.504 | 0.831 | 0.080 |
| Left middle temporal | -0.442 | 0.850 | 0.597 |  | -0.649 | 0.766 | 0.099 |  | -0.699 | 0.753 | 0.108 |  | -0.159 | 0.955 | 0.053 |  | -0.904 | 0.682 | 0.148 |  | 0.084 | 0.976 | 0.051 |  | 0.682 | 0.760 | 0.105 |
| Left parahippocampal | -0.372 | 0.875 | 0.224 |  | 0.377 | 0.874 | 0.066 |  | -0.717 | 0.744 | 0.111 |  | -0.863 | 0.695 | 0.139 |  | -0.869 | 0.693 | 0.140 |  | -1.353 | 0.501 | 0.272 |  | -0.979 | 0.654 | 0.165 |
| Left paracentral | -2.157 | 0.236 | 0.164 |  | -0.147 | 0.957 | 0.052 |  | -0.384 | 0.872 | 0.067 |  | -2.606 | 0.134 | 0.741 |  | -1.653 | 0.387 | 0.380 |  | -2.317 | 0.205 | 0.639 |  | -2.253 | 0.211 | 0.615 |
| Left pars opercularis | 0.170 | 0.952 | 0.242 |  | 0.913 | 0.680 | 0.150 |  | 0.417 | 0.862 | 0.070 |  | -1.181 | 0.568 | 0.219 |  | -1.410 | 0.475 | 0.291 |  | 0.546 | 0.811 | 0.085 |  | -0.929 | 0.677 | 0.153 |
| Left pars orbitalis | -0.255 | 0.919 | 0.053 |  | 0.539 | 0.815 | 0.084 |  | -1.114 | 0.596 | 0.200 |  | 0.101 | 0.970 | 0.051 |  | 0.564 | 0.805 | 0.087 |  | 0.654 | 0.765 | 0.100 |  | 0.149 | 0.957 | 0.053 |
| Left pars triangularis | -1.573 | 0.411 | 0.060 |  | 1.024 | 0.635 | 0.176 |  | -2.128 | 0.241 | 0.567 |  | -2.064 | 0.270 | 0.542 |  | -1.497 | 0.443 | 0.322 |  | -2.555 | 0.144 | 0.724 |  | -2.827 | 0.114 | 0.807 |
| Left pericalcarine | -2.077 | 0.266 | 0.391 |  | -2.149 | 0.236 | 0.575 |  | -1.799 | 0.352 | 0.436 |  | -2.281 | 0.211 | 0.626 |  | -1.974 | 0.300 | 0.506 |  | -1.470 | 0.456 | 0.312 |  | -1.317 | 0.512 | 0.261 |
| Left postcentral | -0.850 | 0.703 | 0.390 |  | -1.608 | 0.394 | 0.363 |  | -3.310 | 0.060 | 0.911 |  | -2.756 | 0.119 | 0.787 |  | -2.416 | 0.178 | 0.676 |  | -1.686 | 0.382 | 0.392 |  | -2.595 | 0.137 | 0.737 |
| Left posterior cingulate | -0.777 | 0.727 | 0.232 |  | -0.240 | 0.923 | 0.057 |  | -0.961 | 0.660 | 0.161 |  | 0.525 | 0.824 | 0.082 |  | -0.919 | 0.680 | 0.151 |  | 0.815 | 0.716 | 0.129 |  | 0.497 | 0.831 | 0.079 |
| Left precentral | -1.709 | 0.374 | 0.225 |  | 0.460 | 0.849 | 0.075 |  | -1.535 | 0.425 | 0.336 |  | -2.275 | 0.211 | 0.624 |  | -1.585 | 0.408 | 0.354 |  | -0.414 | 0.862 | 0.070 |  | -2.421 | 0.178 | 0.678 |
| Left precuneus | -0.609 | 0.788 | 0.128 |  | -0.468 | 0.843 | 0.075 |  | -0.943 | 0.671 | 0.156 |  | -0.732 | 0.738 | 0.113 |  | -1.578 | 0.411 | 0.351 |  | -0.520 | 0.826 | 0.082 |  | -0.471 | 0.841 | 0.076 |
| Left rostral anterior cingulate | -0.009 | 0.996 | 0.505 |  | -2.681 | 0.129 | 0.765 |  | -1.976 | 0.300 | 0.507 |  | -1.457 | 0.457 | 0.308 |  | -2.343 | 0.202 | 0.649 |  | 0.662 | 0.764 | 0.101 |  | 0.039 | 0.995 | 0.050 |
| Left rostral middle frontal | -0.480 | 0.839 | 0.327 |  | - | - | - |  | -1.121 | 0.593 | 0.202 |  | -1.014 | 0.643 | 0.173 |  | -1.899 | 0.322 | 0.476 |  | 0.353 | 0.884 | 0.064 |  | -0.543 | 0.813 | 0.084 |
| Left superior frontal | -0.584 | 0.796 | 0.109 |  | -1.121 | 0.593 | 0.202 |  | - | - | - |  | -2.245 | 0.211 | 0.612 |  | -2.210 | 0.222 | 0.599 |  | 0.010 | 0.996 | 0.050 |  | -0.799 | 0.721 | 0.126 |
| Left superior parietal | -0.430 | 0.855 | 0.069 |  | -0.641 | 0.768 | 0.098 |  | -1.893 | 0.322 | 0.473 |  | -3.026 | 0.077 | 0.857 |  | -2.255 | 0.211 | 0.616 |  | -0.809 | 0.719 | 0.128 |  | -1.901 | 0.322 | 0.477 |
| Left superior temporal | -0.432 | 0.855 | 0.819 |  | -1.014 | 0.643 | 0.173 |  | -2.245 | 0.211 | 0.612 |  | - | - | - |  | -2.647 | 0.129 | 0.754 |  | -1.691 | 0.380 | 0.394 |  | -1.698 | 0.378 | 0.397 |
| Left supramarginal | -0.234 | 0.923 | 0.066 |  | 0.263 | 0.916 | 0.058 |  | -0.581 | 0.797 | 0.090 |  | -2.312 | 0.205 | 0.637 |  | -1.862 | 0.332 | 0.461 |  | -0.344 | 0.885 | 0.064 |  | -1.969 | 0.300 | 0.504 |
| Left frontal pole | -0.267 | 0.915 | 0.073 |  | -1.062 | 0.618 | 0.186 |  | -0.829 | 0.713 | 0.132 |  | -0.884 | 0.689 | 0.143 |  | -1.254 | 0.533 | 0.241 |  | -0.623 | 0.777 | 0.096 |  | -0.291 | 0.912 | 0.060 |
| Left temporal pole | 0.179 | 0.947 | 0.194 |  | -0.316 | 0.900 | 0.061 |  | -0.819 | 0.714 | 0.130 |  | -0.359 | 0.880 | 0.065 |  | -0.954 | 0.664 | 0.159 |  | -1.640 | 0.388 | 0.375 |  | -0.794 | 0.722 | 0.125 |
| Left transverse temporal | -1.393 | 0.479 | 0.081 |  | -1.899 | 0.322 | 0.476 |  | -2.210 | 0.222 | 0.599 |  | -2.647 | 0.129 | 0.754 |  | - | - | - |  | -0.835 | 0.710 | 0.133 |  | -2.315 | 0.205 | 0.639 |
| Left insula | 0.477 | 0.840 | 0.088 |  | 0.711 | 0.744 | 0.110 |  | 0.437 | 0.853 | 0.072 |  | 1.237 | 0.539 | 0.236 |  | -1.092 | 0.603 | 0.194 |  | 0.884 | 0.689 | 0.143 |  | 0.599 | 0.789 | 0.092 |
| Right banks of superior temporal sulcus | -0.664 | 0.763 | 0.065 |  | -0.862 | 0.695 | 0.138 |  | -1.496 | 0.443 | 0.322 |  | -1.157 | 0.579 | 0.212 |  | -0.155 | 0.957 | 0.053 |  | -0.193 | 0.946 | 0.054 |  | -0.300 | 0.909 | 0.060 |
| Right caudal anterior cingulate | 1.111 | 0.596 | 0.062 |  | 1.905 | 0.322 | 0.478 |  | 1.103 | 0.600 | 0.197 |  | 0.547 | 0.811 | 0.085 |  | -0.552 | 0.809 | 0.086 |  | 0.963 | 0.659 | 0.161 |  | -0.167 | 0.953 | 0.053 |
| Right caudal middle frontal | -0.294 | 0.911 | 0.065 |  | 0.383 | 0.872 | 0.067 |  | -1.056 | 0.619 | 0.184 |  | -1.780 | 0.358 | 0.429 |  | -0.253 | 0.919 | 0.057 |  | 0.819 | 0.714 | 0.130 |  | -0.568 | 0.803 | 0.088 |
| Right cuneus | -1.108 | 0.597 | 0.309 |  | -2.193 | 0.225 | 0.592 |  | -0.904 | 0.682 | 0.148 |  | -0.915 | 0.680 | 0.150 |  | -1.996 | 0.295 | 0.515 |  | -1.351 | 0.501 | 0.272 |  | -0.870 | 0.693 | 0.140 |
| Right entorhinal | 0.587 | 0.795 | 0.172 |  | 0.672 | 0.761 | 0.103 |  | 0.179 | 0.947 | 0.054 |  | 0.136 | 0.958 | 0.052 |  | -0.861 | 0.695 | 0.138 |  | 0.011 | 0.996 | 0.050 |  | -0.398 | 0.866 | 0.068 |
| Right fusiform | -0.515 | 0.828 | 0.373 |  | 0.511 | 0.830 | 0.080 |  | -0.081 | 0.977 | 0.051 |  | 0.288 | 0.912 | 0.060 |  | -0.798 | 0.721 | 0.125 |  | -1.369 | 0.491 | 0.278 |  | -0.754 | 0.729 | 0.117 |
| Right inferior parietal | -1.838 | 0.342 | 0.516 |  | 0.402 | 0.865 | 0.069 |  | -1.102 | 0.600 | 0.197 |  | -3.612 | **0.034** | 0.951 |  | -1.724 | 0.372 | 0.407 |  | -0.530 | 0.821 | 0.083 |  | -2.836 | 0.114 | 0.810 |
| Right inferior temporal | -0.475 | 0.840 | 0.050 |  | -0.015 | 0.996 | 0.050 |  | -1.253 | 0.533 | 0.240 |  | -3.262 | 0.060 | 0.904 |  | -1.537 | 0.425 | 0.336 |  | -1.206 | 0.554 | 0.226 |  | -3.437 | 0.052 | 0.930 |
| Right isthmus cingulate | -2.091 | 0.260 | 0.108 |  | -0.784 | 0.727 | 0.123 |  | -1.947 | 0.309 | 0.495 |  | -1.071 | 0.615 | 0.188 |  | -2.637 | 0.129 | 0.751 |  | -1.463 | 0.457 | 0.310 |  | -2.350 | 0.200 | 0.652 |
| Right lateral occipital | -1.639 | 0.388 | 0.081 |  | -0.217 | 0.934 | 0.055 |  | -0.774 | 0.727 | 0.121 |  | -1.860 | 0.332 | 0.460 |  | -2.710 | 0.121 | 0.773 |  | -0.715 | 0.744 | 0.110 |  | -2.309 | 0.205 | 0.637 |
| Right lateral orbitofrontal | 1.759 | 0.359 | 0.764 |  | 0.496 | 0.831 | 0.079 |  | 1.041 | 0.628 | 0.180 |  | 1.189 | 0.564 | 0.221 |  | -0.237 | 0.923 | 0.056 |  | 1.443 | 0.462 | 0.303 |  | 1.334 | 0.504 | 0.266 |
| Right lingual | -1.009 | 0.644 | 0.277 |  | -1.655 | 0.387 | 0.380 |  | -1.575 | 0.411 | 0.350 |  | -0.425 | 0.856 | 0.071 |  | -1.637 | 0.388 | 0.374 |  | -0.341 | 0.886 | 0.063 |  | -0.995 | 0.651 | 0.169 |
| Right medial orbitofrontal | 1.259 | 0.533 | 0.921 |  | 0.905 | 0.682 | 0.148 |  | 0.655 | 0.765 | 0.100 |  | 0.344 | 0.885 | 0.064 |  | 0.303 | 0.909 | 0.061 |  | 0.872 | 0.692 | 0.141 |  | 1.148 | 0.581 | 0.209 |
| Right middle temporal | -1.365 | 0.493 | 0.169 |  | 0.353 | 0.884 | 0.064 |  | 0.010 | 0.996 | 0.050 |  | -1.691 | 0.380 | 0.394 |  | -0.835 | 0.710 | 0.133 |  | - | - | - |  | -1.597 | 0.400 | 0.359 |
| Right parahippocampal | -1.308 | 0.513 | 0.209 |  | 0.396 | 0.867 | 0.068 |  | -0.825 | 0.713 | 0.131 |  | -1.520 | 0.432 | 0.330 |  | -1.282 | 0.525 | 0.249 |  | -2.671 | 0.129 | 0.762 |  | -1.778 | 0.358 | 0.428 |
| Right paracentral | -2.448 | 0.169 | 0.400 |  | -1.638 | 0.388 | 0.374 |  | -2.233 | 0.212 | 0.608 |  | -2.374 | 0.195 | 0.661 |  | -2.559 | 0.144 | 0.725 |  | -1.818 | 0.350 | 0.444 |  | -1.829 | 0.347 | 0.448 |
| Right pars opercularis | -1.221 | 0.551 | 0.055 |  | 0.139 | 0.958 | 0.052 |  | -1.130 | 0.590 | 0.204 |  | -4.709 | **0.002** | 0.997 |  | -1.988 | 0.295 | 0.511 |  | -1.356 | 0.499 | 0.273 |  | -3.907 | **0.028** | 0.974 |
| Right pars orbitalis | 0.573 | 0.801 | 0.112 |  | 0.453 | 0.850 | 0.074 |  | 0.409 | 0.865 | 0.069 |  | -0.165 | 0.954 | 0.053 |  | 1.377 | 0.486 | 0.280 |  | 1.153 | 0.579 | 0.211 |  | 0.212 | 0.936 | 0.055 |
| Right pars triangularis | -0.813 | 0.716 | 0.372 |  | -1.280 | 0.525 | 0.249 |  | -1.740 | 0.362 | 0.413 |  | -1.380 | 0.486 | 0.281 |  | -0.882 | 0.689 | 0.143 |  | -0.349 | 0.885 | 0.064 |  | -1.379 | 0.486 | 0.281 |
| Right pericalcarine | -2.242 | 0.211 | 0.226 |  | -1.611 | 0.394 | 0.364 |  | -1.418 | 0.475 | 0.294 |  | -1.709 | 0.374 | 0.401 |  | -1.406 | 0.475 | 0.290 |  | -0.861 | 0.695 | 0.138 |  | -0.739 | 0.736 | 0.115 |
| Right postcentral | -2.136 | 0.240 | 0.179 |  | -1.434 | 0.463 | 0.300 |  | -3.523 | **0.043** | 0.941 |  | -3.681 | **0.034** | 0.957 |  | -1.759 | 0.359 | 0.421 |  | -2.096 | 0.258 | 0.554 |  | -3.256 | 0.060 | 0.903 |
| Right posterior cingulate | -1.988 | 0.295 | 0.417 |  | -1.209 | 0.554 | 0.227 |  | -1.484 | 0.447 | 0.317 |  | -0.929 | 0.677 | 0.153 |  | -2.151 | 0.236 | 0.576 |  | -1.062 | 0.618 | 0.186 |  | -1.270 | 0.527 | 0.246 |
| Right precentral | -1.775 | 0.358 | 0.073 |  | 0.744 | 0.736 | 0.116 |  | -1.612 | 0.394 | 0.364 |  | -3.063 | 0.073 | 0.865 |  | -1.170 | 0.575 | 0.216 |  | -0.449 | 0.850 | 0.073 |  | -2.726 | 0.121 | 0.778 |
| Right precuneus | -2.144 | 0.237 | 0.050 |  | -1.883 | 0.326 | 0.469 |  | -2.299 | 0.205 | 0.633 |  | -1.213 | 0.554 | 0.228 |  | -2.030 | 0.277 | 0.528 |  | -1.504 | 0.440 | 0.324 |  | -0.501 | 0.831 | 0.079 |
| Right rostral anterior cingulate | 0.572 | 0.801 | 0.453 |  | 0.946 | 0.671 | 0.157 |  | 0.559 | 0.806 | 0.087 |  | 0.559 | 0.806 | 0.087 |  | 0.492 | 0.832 | 0.078 |  | 1.616 | 0.394 | 0.366 |  | 0.647 | 0.766 | 0.099 |
| Right rostral middle frontal | 0.657 | 0.765 | 0.165 |  | -0.123 | 0.963 | 0.052 |  | -0.775 | 0.727 | 0.121 |  | -0.824 | 0.713 | 0.131 |  | -0.492 | 0.832 | 0.078 |  | 1.602 | 0.397 | 0.360 |  | 0.602 | 0.789 | 0.093 |
| Right superior frontal | -1.096 | 0.601 | 0.051 |  | -0.598 | 0.789 | 0.092 |  | -1.244 | 0.537 | 0.238 |  | -1.200 | 0.558 | 0.224 |  | -1.872 | 0.330 | 0.465 |  | 0.286 | 0.912 | 0.059 |  | -0.077 | 0.979 | 0.051 |
| Right superior parietal | -1.707 | 0.374 | 0.069 |  | -1.519 | 0.432 | 0.330 |  | -2.649 | 0.129 | 0.755 |  | -2.743 | 0.121 | 0.783 |  | -2.636 | 0.129 | 0.751 |  | -1.255 | 0.533 | 0.241 |  | -2.208 | 0.222 | 0.598 |
| Right superior temporal | 0.047 | 0.990 | 0.057 |  | -0.543 | 0.813 | 0.084 |  | -0.799 | 0.721 | 0.126 |  | -1.698 | 0.378 | 0.397 |  | -2.315 | 0.205 | 0.639 |  | -1.597 | 0.400 | 0.359 |  | - | - | - |
| Right supramarginal | -2.460 | 0.169 | 0.174 |  | -0.850 | 0.703 | 0.136 |  | -2.267 | 0.211 | 0.621 |  | -3.121 | 0.070 | 0.877 |  | -1.746 | 0.362 | 0.415 |  | -1.804 | 0.352 | 0.438 |  | -3.136 | 0.070 | 0.880 |
| Right frontal pole | 1.154 | 0.579 | 0.208 |  | -0.021 | 0.996 | 0.050 |  | -0.092 | 0.972 | 0.051 |  | 0.246 | 0.923 | 0.057 |  | -0.881 | 0.689 | 0.142 |  | 1.113 | 0.596 | 0.200 |  | 0.769 | 0.728 | 0.120 |
| Right temporal pole | -0.913 | 0.680 | 0.054 |  | -0.737 | 0.736 | 0.114 |  | -1.337 | 0.504 | 0.267 |  | -1.629 | 0.391 | 0.370 |  | -2.498 | 0.160 | 0.705 |  | -2.459 | 0.169 | 0.691 |  | -1.865 | 0.332 | 0.462 |
| Right transverse temporal | -0.759 | 0.728 | 0.115 |  | -0.099 | 0.970 | 0.051 |  | -1.274 | 0.526 | 0.247 |  | -2.760 | 0.119 | 0.788 |  | -1.544 | 0.424 | 0.339 |  | -1.096 | 0.601 | 0.195 |  | -2.190 | 0.225 | 0.591 |
| Right insula | -0.037 | 0.995 | 0.180 |  | -2.069 | 0.270 | 0.543 |  | -0.393 | 0.868 | 0.068 |  | -0.911 | 0.681 | 0.149 |  | -1.892 | 0.322 | 0.473 |  | 1.121 | 0.593 | 0.202 |  | -0.667 | 0.763 | 0.102 |

Abbreviations: BD-I, bipolar disorder type I; HCs, healthy subject controls; z, between-group z statistic (BD-I vs HCs).

The p values are presented between-group test after FDR correction for all comparisons.

Bold values indicate significant differences with p < 0.05.

**Table S3.** Structural covariance differences of BD-II vs HCs.

| Structural covariance connectivity | Left inferior temporal | | |  | Left rostralmiddle frontal | | |  | Left superior frontal | | |  | Left superior temporal | | |  | Left transverse temporal | | |  | Right middle temporal | | |  | Right superior temporal | | |
| --- | --- | --- | --- | --- | --- | --- | --- | --- | --- | --- | --- | --- | --- | --- | --- | --- | --- | --- | --- | --- | --- | --- | --- | --- | --- | --- | --- |
|  | z | p | Power |  | z | p | Power |  | z | p | Power |  | z | p | Power |  | z | p | Power |  | z | p | Power |  | z | p | Power |
| Left banks of superior temporal sulcus | 0.628 | 0.775 | 0.096 |  | 0.664 | 0.763 | 0.102 |  | 0.549 | 0.811 | 0.085 |  | 0.269 | 0.915 | 0.058 |  | -0.834 | 0.710 | 0.133 |  | 1.654 | 0.387 | 0.380 |  | -0.497 | 0.831 | 0.079 |
| Left caudal anterior cingulate | -0.836 | 0.710 | 0.133 |  | -0.764 | 0.728 | 0.119 |  | -0.426 | 0.856 | 0.071 |  | -0.987 | 0.652 | 0.167 |  | -2.505 | 0.159 | 0.707 |  | -1.006 | 0.644 | 0.171 |  | -1.796 | 0.353 | 0.435 |
| Left caudal middle frontal | 0.268 | 0.915 | 0.058 |  | -0.007 | 0.996 | 0.050 |  | -1.691 | 0.380 | 0.394 |  | -0.763 | 0.728 | 0.119 |  | -1.438 | 0.463 | 0.301 |  | -0.100 | 0.970 | 0.051 |  | -0.763 | 0.728 | 0.119 |
| Left cuneus | -1.740 | 0.362 | 0.413 |  | -1.945 | 0.309 | 0.494 |  | -1.282 | 0.525 | 0.250 |  | -1.863 | 0.332 | 0.461 |  | -0.839 | 0.710 | 0.134 |  | -1.570 | 0.412 | 0.348 |  | -0.795 | 0.722 | 0.125 |
| Left entorhinal | 0.649 | 0.766 | 0.099 |  | 0.758 | 0.728 | 0.118 |  | 0.282 | 0.913 | 0.059 |  | 1.408 | 0.475 | 0.291 |  | 0.260 | 0.918 | 0.058 |  | 1.090 | 0.604 | 0.193 |  | -0.209 | 0.936 | 0.055 |
| Left fusiform | 0.563 | 0.805 | 0.087 |  | 1.562 | 0.415 | 0.346 |  | 1.291 | 0.524 | 0.252 |  | 0.304 | 0.909 | 0.061 |  | -1.124 | 0.593 | 0.203 |  | -1.146 | 0.582 | 0.209 |  | -0.998 | 0.650 | 0.170 |
| Left inferior parietal | 0.034 | 0.995 | 0.050 |  | -1.063 | 0.618 | 0.186 |  | -2.182 | 0.227 | 0.588 |  | -2.058 | 0.270 | 0.539 |  | -1.678 | 0.384 | 0.389 |  | 0.211 | 0.936 | 0.055 |  | -0.891 | 0.689 | 0.145 |
| Left inferior temporal | - | - | - |  | 0.925 | 0.679 | 0.152 |  | 0.113 | 0.966 | 0.051 |  | 2.188 | 0.225 | 0.590 |  | -1.711 | 0.374 | 0.402 |  | -0.332 | 0.891 | 0.063 |  | -0.173 | 0.951 | 0.053 |
| Left isthmus cingulate | 0.017 | 0.996 | 0.050 |  | -0.916 | 0.680 | 0.150 |  | -0.594 | 0.790 | 0.091 |  | 0.489 | 0.833 | 0.078 |  | -0.738 | 0.736 | 0.114 |  | 0.407 | 0.865 | 0.069 |  | 0.273 | 0.915 | 0.059 |
| Left lateral occipital | -1.760 | 0.359 | 0.421 |  | -1.670 | 0.386 | 0.386 |  | -1.481 | 0.449 | 0.316 |  | -2.532 | 0.149 | 0.716 |  | -0.522 | 0.825 | 0.082 |  | -1.458 | 0.457 | 0.308 |  | -1.339 | 0.504 | 0.268 |
| Left lateral orbitofrontal | -0.108 | 0.969 | 0.051 |  | -2.239 | 0.211 | 0.610 |  | 0.024 | 0.996 | 0.050 |  | 0.539 | 0.815 | 0.084 |  | -1.467 | 0.457 | 0.311 |  | 0.822 | 0.714 | 0.130 |  | -1.152 | 0.579 | 0.211 |
| Left lingual | -0.134 | 0.958 | 0.052 |  | -0.223 | 0.930 | 0.056 |  | 0.283 | 0.913 | 0.059 |  | -0.802 | 0.719 | 0.126 |  | -0.454 | 0.850 | 0.074 |  | -0.234 | 0.923 | 0.056 |  | -0.251 | 0.920 | 0.057 |
| Left medial orbitofrontal | -2.055 | 0.270 | 0.538 |  | -1.262 | 0.532 | 0.243 |  | -1.843 | 0.342 | 0.453 |  | -0.756 | 0.728 | 0.118 |  | 0.607 | 0.788 | 0.093 |  | -0.989 | 0.652 | 0.167 |  | 0.281 | 0.913 | 0.059 |
| Left middle temporal | 1.584 | 0.408 | 0.354 |  | 0.449 | 0.850 | 0.073 |  | 0.358 | 0.880 | 0.065 |  | 0.605 | 0.788 | 0.093 |  | -1.770 | 0.359 | 0.425 |  | 0.814 | 0.716 | 0.129 |  | 0.259 | 0.918 | 0.058 |
| Left parahippocampal | 0.743 | 0.736 | 0.115 |  | 1.013 | 0.643 | 0.173 |  | 0.064 | 0.982 | 0.050 |  | 0.153 | 0.957 | 0.053 |  | -0.114 | 0.966 | 0.051 |  | 0.723 | 0.742 | 0.112 |  | 0.056 | 0.984 | 0.050 |
| Left paracentral | -1.060 | 0.619 | 0.185 |  | -0.722 | 0.742 | 0.112 |  | -0.877 | 0.689 | 0.142 |  | -1.302 | 0.517 | 0.256 |  | -0.682 | 0.760 | 0.105 |  | -1.815 | 0.350 | 0.443 |  | -0.414 | 0.862 | 0.070 |
| Left pars opercularis | 1.281 | 0.525 | 0.249 |  | 0.697 | 0.753 | 0.107 |  | 0.274 | 0.915 | 0.059 |  | -0.768 | 0.728 | 0.120 |  | -2.033 | 0.277 | 0.529 |  | 1.270 | 0.527 | 0.246 |  | -2.244 | 0.211 | 0.612 |
| Left pars orbitalis | -0.076 | 0.979 | 0.051 |  | -0.474 | 0.840 | 0.076 |  | 0.058 | 0.984 | 0.050 |  | 0.087 | 0.975 | 0.051 |  | -0.032 | 0.995 | 0.050 |  | 1.330 | 0.504 | 0.265 |  | 0.106 | 0.969 | 0.051 |
| Left pars triangularis | -1.143 | 0.582 | 0.208 |  | 0.142 | 0.957 | 0.052 |  | -1.536 | 0.425 | 0.336 |  | -1.183 | 0.568 | 0.219 |  | -0.735 | 0.736 | 0.114 |  | -3.096 | 0.070 | 0.872 |  | -3.079 | 0.072 | 0.868 |
| Left pericalcarine | -3.375 | 0.055 | 0.922 |  | -2.710 | 0.121 | 0.773 |  | -2.453 | 0.169 | 0.689 |  | -2.874 | 0.107 | 0.820 |  | -1.714 | 0.374 | 0.403 |  | -2.914 | 0.097 | 0.830 |  | -1.454 | 0.457 | 0.307 |
| Left postcentral | -2.272 | 0.211 | 0.623 |  | -2.802 | 0.114 | 0.800 |  | -3.708 | **0.034** | 0.960 |  | -3.255 | 0.060 | 0.902 |  | -1.624 | 0.391 | 0.369 |  | -2.365 | 0.195 | 0.657 |  | -1.801 | 0.352 | 0.437 |
| Left posterior cingulate | 0.403 | 0.865 | 0.069 |  | -0.294 | 0.911 | 0.060 |  | -0.243 | 0.923 | 0.057 |  | 0.725 | 0.742 | 0.112 |  | -0.359 | 0.880 | 0.065 |  | 1.656 | 0.387 | 0.381 |  | 0.974 | 0.654 | 0.164 |
| Left precentral | -0.455 | 0.850 | 0.074 |  | -0.714 | 0.744 | 0.110 |  | -2.030 | 0.277 | 0.528 |  | -0.584 | 0.796 | 0.090 |  | -1.811 | 0.351 | 0.441 |  | -0.890 | 0.689 | 0.145 |  | -1.152 | 0.579 | 0.211 |
| Left precuneus | 0.181 | 0.947 | 0.054 |  | -0.976 | 0.654 | 0.164 |  | -0.701 | 0.752 | 0.108 |  | -1.240 | 0.538 | 0.236 |  | -1.242 | 0.538 | 0.237 |  | 0.119 | 0.964 | 0.052 |  | -0.270 | 0.915 | 0.058 |
| Left rostral anterior cingulate | -1.779 | 0.358 | 0.428 |  | -2.917 | 0.097 | 0.831 |  | -2.052 | 0.270 | 0.537 |  | -1.951 | 0.309 | 0.496 |  | -1.957 | 0.307 | 0.499 |  | -0.804 | 0.719 | 0.127 |  | -1.036 | 0.628 | 0.179 |
| Left rostral middle frontal | 0.925 | 0.679 | 0.152 |  | - | - | - |  | -0.993 | 0.652 | 0.168 |  | 0.095 | 0.971 | 0.051 |  | -1.630 | 0.391 | 0.371 |  | 0.500 | 0.831 | 0.079 |  | -1.202 | 0.557 | 0.225 |
| Left superior frontal | 0.113 | 0.966 | 0.051 |  | -0.993 | 0.652 | 0.168 |  | - | - | - |  | -1.549 | 0.423 | 0.341 |  | -2.786 | 0.114 | 0.795 |  | 0.555 | 0.809 | 0.086 |  | -1.426 | 0.469 | 0.297 |
| Left superior parietal | -0.019 | 0.996 | 0.050 |  | -1.333 | 0.504 | 0.266 |  | -3.185 | 0.069 | 0.890 |  | -2.306 | 0.205 | 0.635 |  | -0.837 | 0.710 | 0.133 |  | -0.671 | 0.761 | 0.103 |  | -1.025 | 0.635 | 0.176 |
| Left superior temporal | 2.188 | 0.225 | 0.590 |  | 0.095 | 0.971 | 0.051 |  | -1.549 | 0.423 | 0.341 |  | - | - | - |  | -2.807 | 0.114 | 0.802 |  | 0.022 | 0.996 | 0.050 |  | -1.791 | 0.355 | 0.433 |
| Left supramarginal | 0.119 | 0.964 | 0.052 |  | -0.181 | 0.947 | 0.054 |  | -0.484 | 0.836 | 0.077 |  | -0.721 | 0.742 | 0.111 |  | -1.550 | 0.423 | 0.341 |  | -0.450 | 0.850 | 0.073 |  | -1.085 | 0.608 | 0.192 |
| Left frontal pole | -0.643 | 0.767 | 0.099 |  | -1.759 | 0.359 | 0.421 |  | -0.738 | 0.736 | 0.114 |  | -0.644 | 0.767 | 0.099 |  | 0.030 | 0.995 | 0.050 |  | -0.184 | 0.947 | 0.054 |  | 0.066 | 0.982 | 0.050 |
| Left temporal pole | 1.143 | 0.582 | 0.208 |  | 0.904 | 0.682 | 0.148 |  | 0.774 | 0.727 | 0.121 |  | 0.324 | 0.895 | 0.062 |  | -1.228 | 0.546 | 0.233 |  | 0.244 | 0.923 | 0.057 |  | -1.441 | 0.462 | 0.302 |
| Left transverse temporal | -1.711 | 0.374 | 0.402 |  | -1.630 | 0.391 | 0.371 |  | -2.786 | 0.114 | 0.795 |  | -2.807 | 0.114 | 0.802 |  | - | - | - |  | -1.640 | 0.388 | 0.375 |  | -3.256 | 0.060 | 0.902 |
| Left insula | -0.086 | 0.975 | 0.051 |  | 0.692 | 0.755 | 0.106 |  | 0.677 | 0.761 | 0.104 |  | 0.684 | 0.760 | 0.105 |  | -0.367 | 0.878 | 0.066 |  | 0.680 | 0.761 | 0.104 |  | -0.180 | 0.947 | 0.054 |
| Right banks of superior temporal sulcus | -0.274 | 0.915 | 0.059 |  | -0.448 | 0.850 | 0.073 |  | -0.238 | 0.923 | 0.057 |  | -1.152 | 0.579 | 0.211 |  | -2.647 | 0.129 | 0.754 |  | 0.825 | 0.713 | 0.131 |  | -0.889 | 0.689 | 0.144 |
| Right caudal anterior cingulate | 1.286 | 0.525 | 0.251 |  | 1.409 | 0.475 | 0.291 |  | 2.311 | 0.205 | 0.637 |  | 0.727 | 0.741 | 0.112 |  | 1.341 | 0.504 | 0.268 |  | 1.609 | 0.394 | 0.363 |  | 0.141 | 0.957 | 0.052 |
| Right caudal middle frontal | 0.057 | 0.984 | 0.050 |  | -0.514 | 0.828 | 0.081 |  | -0.605 | 0.788 | 0.093 |  | -0.968 | 0.659 | 0.162 |  | -0.240 | 0.923 | 0.057 |  | 0.735 | 0.736 | 0.114 |  | 0.183 | 0.947 | 0.054 |
| Right cuneus | -2.306 | 0.205 | 0.635 |  | -2.628 | 0.131 | 0.748 |  | -1.510 | 0.437 | 0.327 |  | -1.992 | 0.295 | 0.513 |  | -0.939 | 0.673 | 0.155 |  | -1.677 | 0.384 | 0.389 |  | -0.676 | 0.761 | 0.104 |
| Right entorhinal | -0.378 | 0.874 | 0.066 |  | -0.073 | 0.979 | 0.051 |  | -0.031 | 0.995 | 0.050 |  | -0.761 | 0.728 | 0.119 |  | -1.627 | 0.391 | 0.370 |  | -0.134 | 0.958 | 0.052 |  | -2.474 | 0.169 | 0.696 |
| Right fusiform | 1.007 | 0.644 | 0.172 |  | 1.182 | 0.568 | 0.219 |  | 1.411 | 0.475 | 0.292 |  | 0.163 | 0.955 | 0.053 |  | -1.040 | 0.628 | 0.180 |  | 0.288 | 0.912 | 0.060 |  | -1.540 | 0.425 | 0.338 |
| Right inferior parietal | 0.146 | 0.957 | 0.052 |  | 0.518 | 0.827 | 0.081 |  | -0.267 | 0.915 | 0.058 |  | -1.037 | 0.628 | 0.179 |  | -1.528 | 0.430 | 0.333 |  | -0.346 | 0.885 | 0.064 |  | -1.259 | 0.533 | 0.242 |
| Right inferior temporal | -0.474 | 0.840 | 0.076 |  | 0.359 | 0.880 | 0.065 |  | -1.399 | 0.477 | 0.288 |  | -2.258 | 0.211 | 0.617 |  | -0.804 | 0.719 | 0.127 |  | -1.914 | 0.322 | 0.482 |  | -2.989 | 0.084 | 0.848 |
| Right isthmus cingulate | -1.248 | 0.536 | 0.239 |  | -0.633 | 0.774 | 0.097 |  | -1.071 | 0.615 | 0.188 |  | -1.452 | 0.457 | 0.306 |  | -1.569 | 0.412 | 0.348 |  | -0.943 | 0.671 | 0.156 |  | -1.015 | 0.643 | 0.174 |
| Right lateral occipital | -1.937 | 0.311 | 0.491 |  | -1.209 | 0.554 | 0.227 |  | -1.214 | 0.554 | 0.229 |  | -2.060 | 0.270 | 0.540 |  | -1.825 | 0.348 | 0.447 |  | -1.252 | 0.533 | 0.240 |  | -1.435 | 0.463 | 0.300 |
| Right lateral orbitofrontal | -0.825 | 0.713 | 0.131 |  | -0.777 | 0.727 | 0.121 |  | 0.098 | 0.970 | 0.051 |  | -0.206 | 0.938 | 0.055 |  | -0.194 | 0.946 | 0.054 |  | -0.805 | 0.719 | 0.127 |  | -0.500 | 0.831 | 0.079 |
| Right lingual | -2.133 | 0.240 | 0.569 |  | -1.757 | 0.359 | 0.420 |  | -0.988 | 0.652 | 0.167 |  | -1.818 | 0.350 | 0.444 |  | -1.522 | 0.432 | 0.331 |  | -1.332 | 0.504 | 0.265 |  | -1.710 | 0.374 | 0.402 |
| Right medial orbitofrontal | -1.895 | 0.322 | 0.474 |  | -1.902 | 0.322 | 0.477 |  | -0.257 | 0.918 | 0.058 |  | -0.537 | 0.815 | 0.084 |  | -0.023 | 0.996 | 0.050 |  | -0.929 | 0.677 | 0.153 |  | 0.129 | 0.961 | 0.052 |
| Right middle temporal | -0.332 | 0.891 | 0.063 |  | 0.500 | 0.831 | 0.079 |  | 0.555 | 0.809 | 0.086 |  | 0.022 | 0.996 | 0.050 |  | -1.640 | 0.388 | 0.375 |  | - | - | - |  | -1.653 | 0.387 | 0.379 |
| Right parahippocampal | -0.143 | 0.957 | 0.052 |  | 0.649 | 0.766 | 0.099 |  | 0.352 | 0.884 | 0.064 |  | -0.877 | 0.689 | 0.142 |  | -1.747 | 0.362 | 0.416 |  | -0.761 | 0.728 | 0.118 |  | -1.804 | 0.352 | 0.438 |
| Right paracentral | -0.666 | 0.763 | 0.102 |  | -1.276 | 0.526 | 0.248 |  | -1.655 | 0.387 | 0.380 |  | -0.924 | 0.679 | 0.152 |  | -2.575 | 0.142 | 0.731 |  | -0.838 | 0.710 | 0.134 |  | -0.695 | 0.754 | 0.107 |
| Right pars opercularis | -1.276 | 0.526 | 0.248 |  | -0.391 | 0.868 | 0.068 |  | -1.041 | 0.628 | 0.180 |  | -3.616 | **0.034** | 0.951 |  | -2.009 | 0.289 | 0.520 |  | -1.684 | 0.382 | 0.392 |  | -3.042 | 0.075 | 0.860 |
| Right pars orbitalis | 1.164 | 0.578 | 0.214 |  | 1.057 | 0.619 | 0.185 |  | 1.317 | 0.512 | 0.261 |  | 0.502 | 0.831 | 0.079 |  | 0.976 | 0.654 | 0.164 |  | 1.129 | 0.591 | 0.204 |  | -0.145 | 0.957 | 0.052 |
| Right pars triangularis | -2.197 | 0.225 | 0.594 |  | -2.793 | 0.114 | 0.797 |  | -2.049 | 0.270 | 0.536 |  | -2.372 | 0.195 | 0.660 |  | -0.012 | 0.996 | 0.050 |  | -1.698 | 0.378 | 0.397 |  | -1.922 | 0.320 | 0.485 |
| Right pericalcarine | -3.095 | 0.070 | 0.872 |  | -2.723 | 0.121 | 0.777 |  | -1.942 | 0.309 | 0.493 |  | -2.260 | 0.211 | 0.618 |  | -0.328 | 0.892 | 0.062 |  | -1.971 | 0.300 | 0.504 |  | -0.072 | 0.979 | 0.051 |
| Right postcentral | -2.847 | 0.113 | 0.813 |  | -3.182 | 0.069 | 0.889 |  | -4.140 | **0.016** | 0.985 |  | -3.761 | **0.034** | 0.964 |  | -1.893 | 0.322 | 0.474 |  | -2.791 | 0.114 | 0.797 |  | -1.739 | 0.362 | 0.413 |
| Right posterior cingulate | -0.215 | 0.934 | 0.055 |  | -0.594 | 0.790 | 0.091 |  | -0.782 | 0.727 | 0.122 |  | -0.416 | 0.862 | 0.070 |  | -1.160 | 0.579 | 0.213 |  | 0.880 | 0.689 | 0.142 |  | -0.766 | 0.728 | 0.119 |
| Right precentral | -1.195 | 0.561 | 0.223 |  | -0.983 | 0.653 | 0.166 |  | -1.456 | 0.457 | 0.307 |  | -2.364 | 0.195 | 0.657 |  | -1.759 | 0.359 | 0.421 |  | -1.207 | 0.554 | 0.227 |  | -1.025 | 0.635 | 0.176 |
| Right precuneus | -1.875 | 0.330 | 0.466 |  | -3.416 | 0.052 | 0.927 |  | -3.130 | 0.070 | 0.879 |  | -2.300 | 0.205 | 0.633 |  | -1.488 | 0.447 | 0.319 |  | -1.219 | 0.551 | 0.230 |  | -0.099 | 0.970 | 0.051 |
| Right rostral anterior cingulate | -1.139 | 0.583 | 0.207 |  | -1.038 | 0.628 | 0.180 |  | -0.113 | 0.966 | 0.051 |  | -0.610 | 0.788 | 0.094 |  | 0.893 | 0.689 | 0.145 |  | 0.178 | 0.947 | 0.054 |  | -0.782 | 0.727 | 0.122 |
| Right rostral middle frontal | -0.290 | 0.912 | 0.060 |  | -2.449 | 0.169 | 0.688 |  | -1.164 | 0.578 | 0.214 |  | -0.954 | 0.664 | 0.159 |  | 0.192 | 0.946 | 0.054 |  | 1.077 | 0.612 | 0.190 |  | -0.145 | 0.957 | 0.052 |
| Right superior frontal | -1.079 | 0.611 | 0.190 |  | -1.916 | 0.322 | 0.482 |  | -1.314 | 0.512 | 0.260 |  | -1.485 | 0.447 | 0.318 |  | -1.675 | 0.384 | 0.388 |  | 0.133 | 0.958 | 0.052 |  | -0.554 | 0.809 | 0.086 |
| Right superior parietal | -1.173 | 0.574 | 0.217 |  | -2.655 | 0.129 | 0.757 |  | -2.979 | 0.084 | 0.846 |  | -2.257 | 0.211 | 0.617 |  | -1.652 | 0.387 | 0.379 |  | -0.659 | 0.765 | 0.101 |  | -0.399 | 0.866 | 0.068 |
| Right superior temporal | -0.173 | 0.951 | 0.053 |  | -1.202 | 0.557 | 0.225 |  | -1.426 | 0.469 | 0.297 |  | -1.791 | 0.355 | 0.433 |  | -3.256 | 0.060 | 0.902 |  | -1.653 | 0.387 | 0.379 |  | - | - | - |
| Right supramarginal | -1.297 | 0.521 | 0.254 |  | -1.312 | 0.512 | 0.259 |  | -1.780 | 0.358 | 0.429 |  | -1.381 | 0.486 | 0.282 |  | -1.398 | 0.477 | 0.288 |  | -1.405 | 0.475 | 0.290 |  | -0.989 | 0.652 | 0.167 |
| Right frontal pole | 0.010 | 0.996 | 0.050 |  | -0.964 | 0.659 | 0.161 |  | 0.432 | 0.855 | 0.072 |  | 0.147 | 0.957 | 0.052 |  | -0.366 | 0.878 | 0.065 |  | 0.975 | 0.654 | 0.164 |  | -0.749 | 0.733 | 0.116 |
| Right temporal pole | -0.655 | 0.765 | 0.100 |  | -0.059 | 0.984 | 0.050 |  | 0.183 | 0.947 | 0.054 |  | -1.345 | 0.503 | 0.270 |  | -2.438 | 0.172 | 0.684 |  | -1.010 | 0.644 | 0.173 |  | -1.285 | 0.525 | 0.250 |
| Right transverse temporal | -1.347 | 0.503 | 0.270 |  | -0.623 | 0.777 | 0.096 |  | -0.762 | 0.728 | 0.119 |  | -2.533 | 0.149 | 0.717 |  | -0.674 | 0.761 | 0.103 |  | -1.448 | 0.459 | 0.305 |  | -1.666 | 0.387 | 0.385 |
| Right insula | -0.966 | 0.659 | 0.162 |  | -2.567 | 0.143 | 0.728 |  | -0.880 | 0.689 | 0.142 |  | -0.922 | 0.679 | 0.152 |  | -0.215 | 0.934 | 0.055 |  | 0.380 | 0.874 | 0.067 |  | -0.402 | 0.865 | 0.069 |

Abbreviations: BD-II, bipolar disorder type II; HCs, healthy subject controls; z, between-group z statistic (BD-II vs HCs).

The p values are presented between-group test after FDR correction for all comparisons.

Bold values indicate significant differences with p < 0.05.


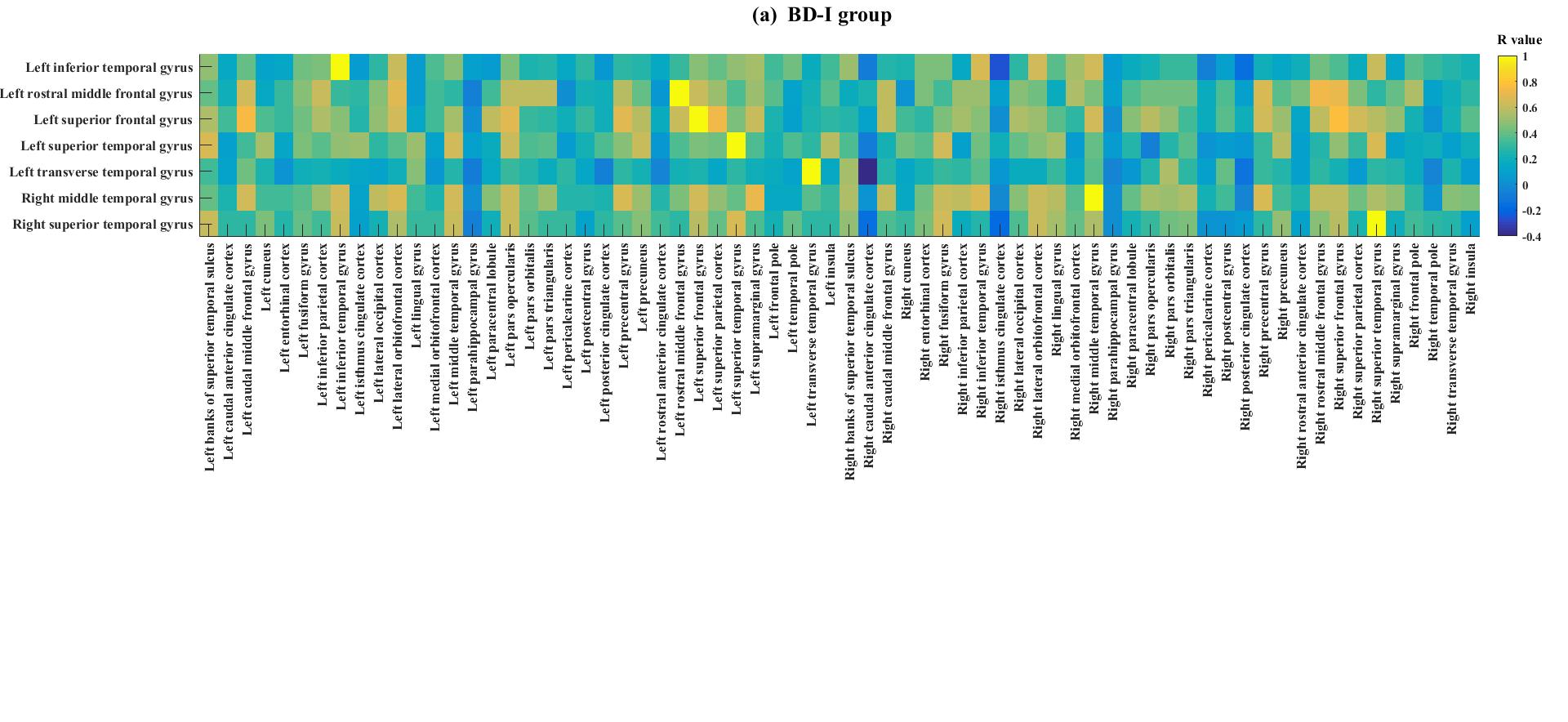


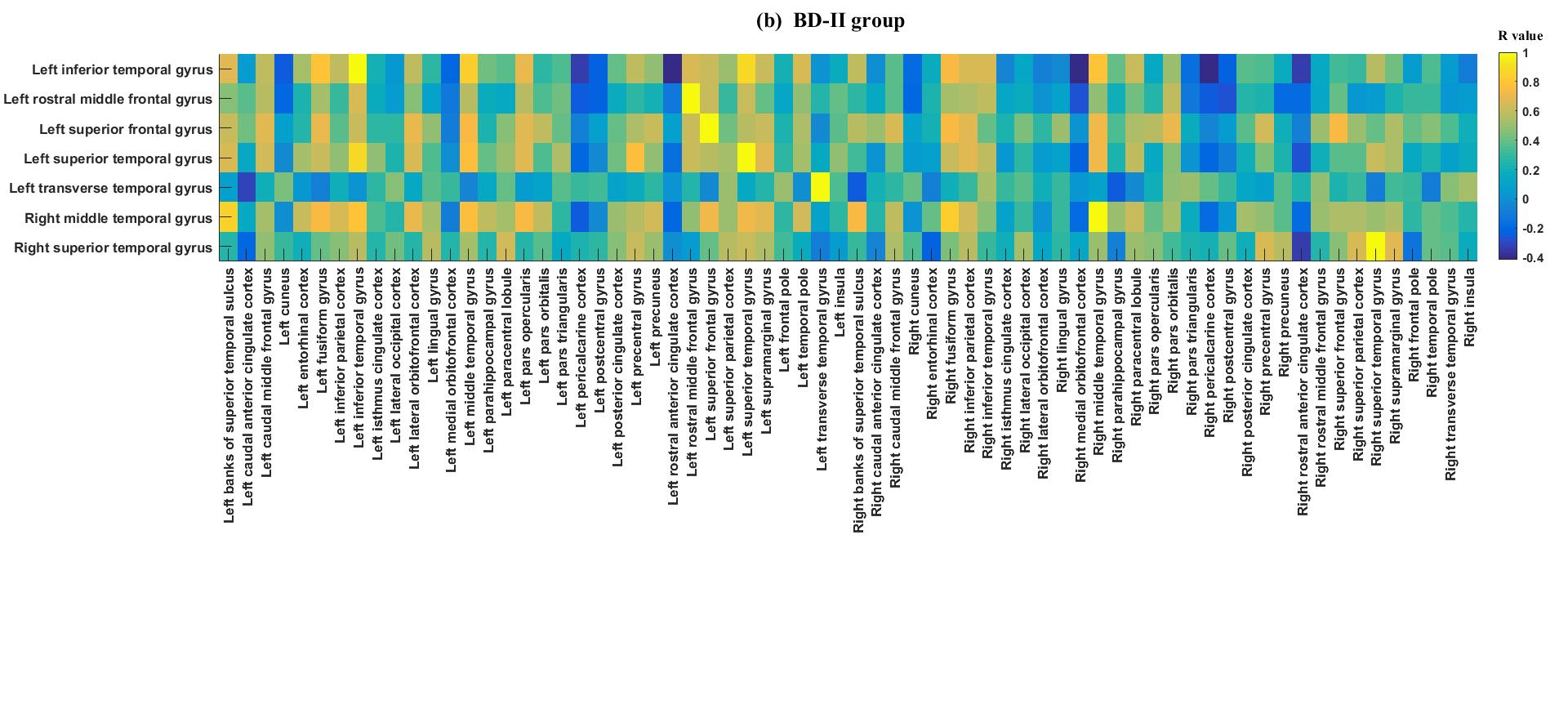


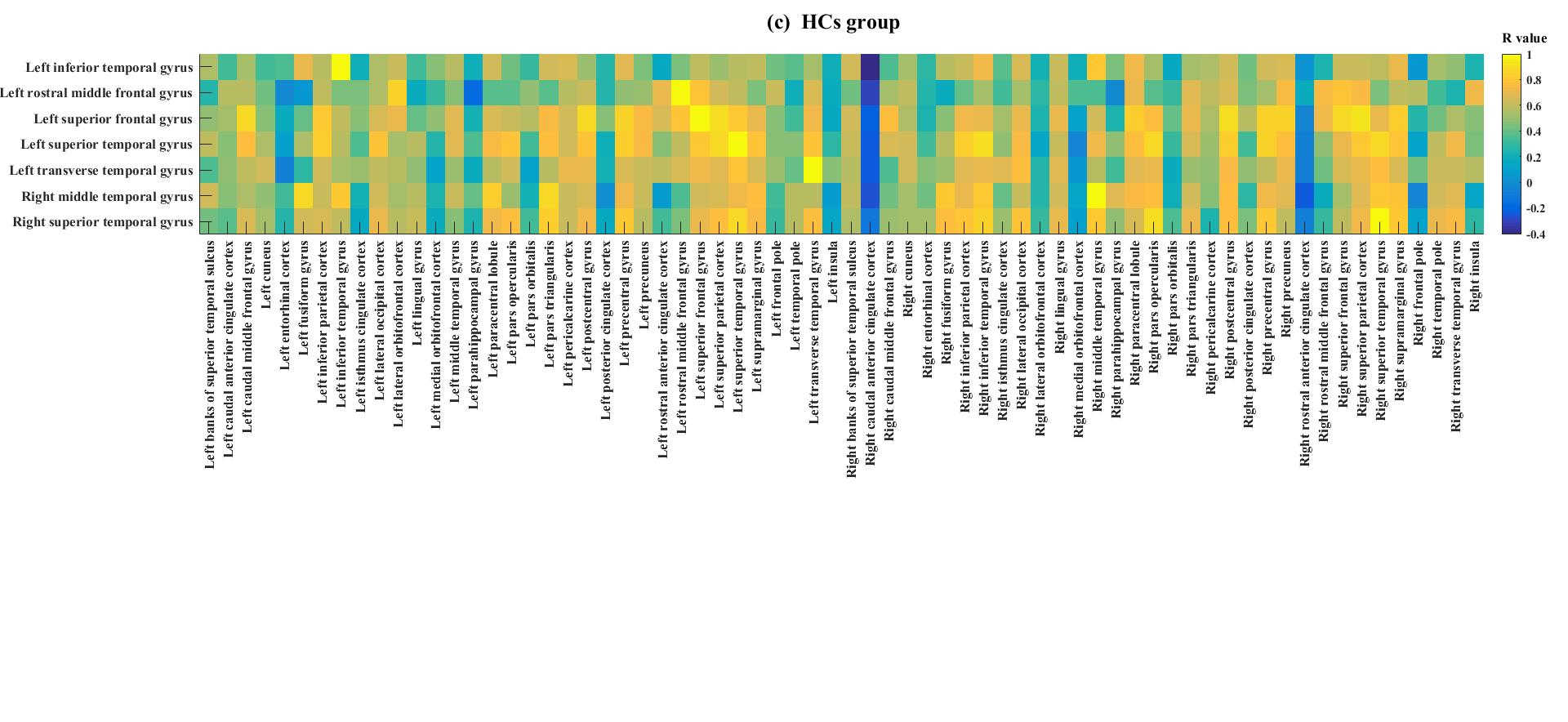


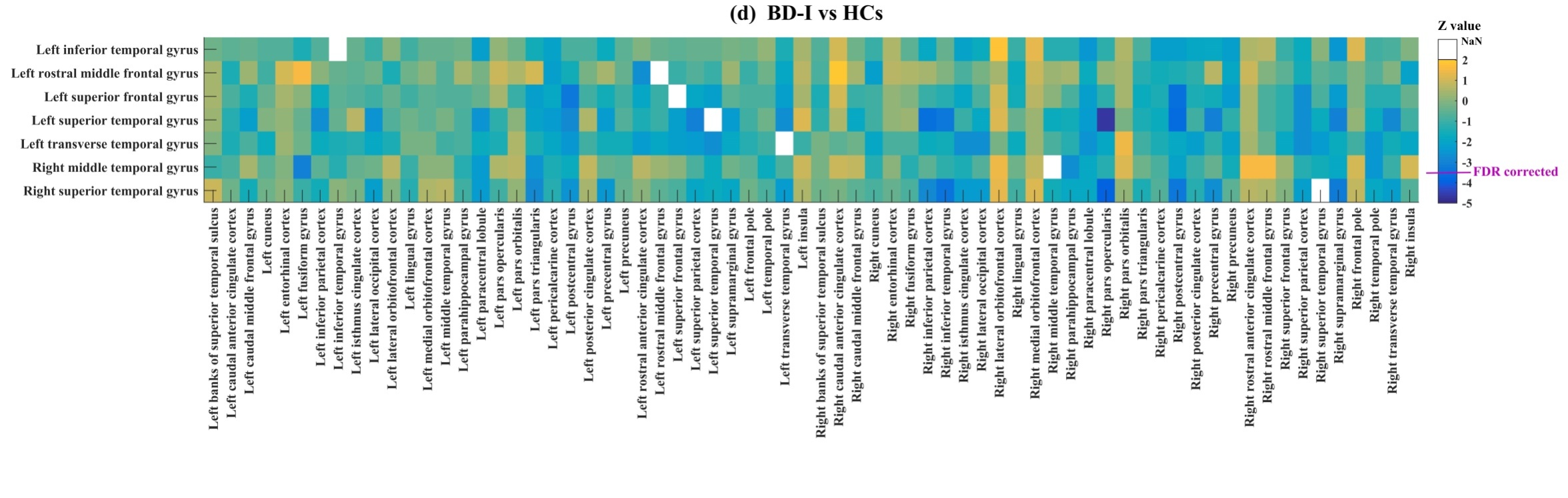


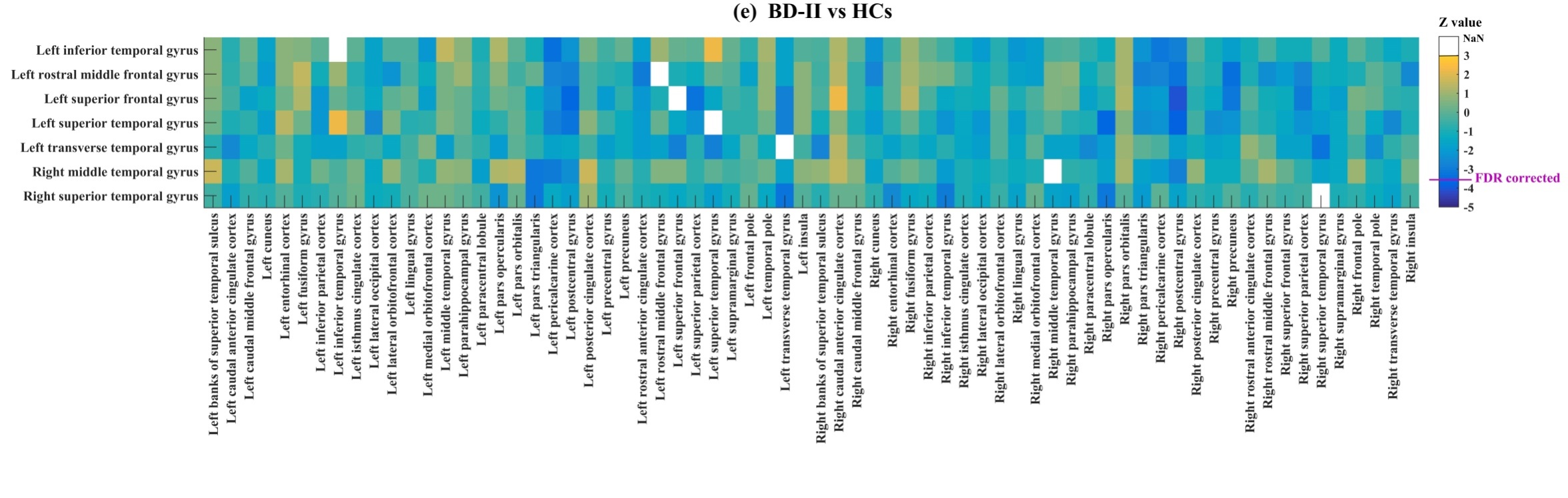


**
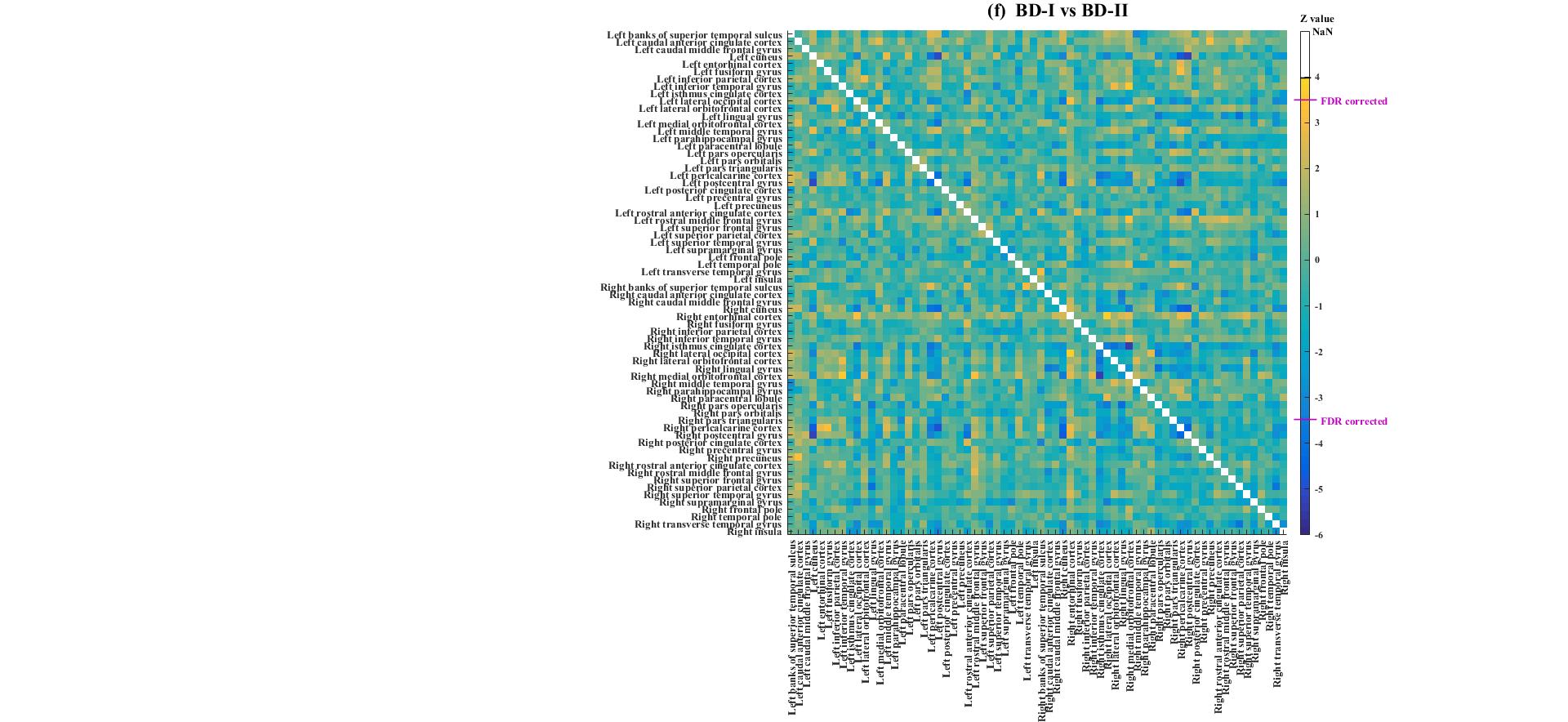
**

**Figure S1.** Cortical structural covariance networks and between-group differences. Figure a, b and c show cortical structural covariance networks of BD-I, BD-II and HCs groups. The color bar shows correlation coefficient.Figure d, e andf show between-group differences (BD-I vs HCs; BD-II vs HCs; BD-I vs BD-II) in cortical structural covariance networks.The color bar shows z value from ztest. Each line ofFDR correctedon color bar represents significantthreshold (p<0.05).In structural covariance networks of Figure d, e and f, white colored gridsrepresentz valueofNaN.
